# A Central Regulatory System Largely Controls Transcriptional Activation and Repression Responses to Phosphate Starvation in Arabidopsis

**DOI:** 10.1371/journal.pgen.1001102

**Published:** 2010-09-09

**Authors:** Regla Bustos, Gabriel Castrillo, Francisco Linhares, María Isabel Puga, Vicente Rubio, Julian Pérez-Pérez, Roberto Solano, Antonio Leyva, Javier Paz-Ares

**Affiliations:** Department of Plant Molecular Genetics, Centro Nacional de Biotecnología-CSIC, Madrid, Spain; The Salk Institute for Biological Studies, United States of America

## Abstract

Plants respond to different stresses by inducing or repressing transcription of partially overlapping sets of genes. In Arabidopsis, the PHR1 transcription factor (TF) has an important role in the control of phosphate (Pi) starvation stress responses. Using transcriptomic analysis of Pi starvation in *phr1*, and *phr1 phr1-like (phl1)* mutants and in wild type plants, we show that *PHR1* in conjunction with *PHL1* controls most transcriptional activation and repression responses to phosphate starvation, regardless of the Pi starvation specificity of these responses. Induced genes are enriched in PHR1 binding sequences (P1BS) in their promoters, whereas repressed genes do not show such enrichment, suggesting that PHR1(-like) control of transcriptional repression responses is indirect. In agreement with this, transcriptomic analysis of a transgenic plant expressing PHR1 fused to the hormone ligand domain of the glucocorticoid receptor showed that PHR1 direct targets (i.e., displaying altered expression after GR:PHR1 activation by dexamethasone in the presence of cycloheximide) corresponded largely to Pi starvation-induced genes that are highly enriched in P1BS. A minimal promoter containing a multimerised P1BS recapitulates Pi starvation-specific responsiveness. Likewise, mutation of P1BS in the promoter of two Pi starvation-responsive genes impaired their responsiveness to Pi starvation, but not to other stress types. Phylogenetic footprinting confirmed the importance of P1BS and PHR1 in Pi starvation responsiveness and indicated that P1BS acts in concert with other *cis* motifs. All together, our data show that PHR1 and PHL1 are partially redundant TF acting as central integrators of Pi starvation responses, both specific and generic. In addition, they indicate that transcriptional repression responses are an integral part of adaptive responses to stress.

## Author Summary

As sessile organisms, plants are often exposed to stress conditions, and have evolved adaptive responses to protect themselves from different types of stress. Some responses are stress type-specific whereas others are common to different stress types. Understanding how these responses are controlled is crucial for rational improvement of stress tolerance, a limiting factor in crop productivity. Here we examined the physiological and molecular responses to phosphate starvation and found that a single transcription factor family, represented by PHOSPHATE STARVATION RESPONSE REGULATOR 1 (PHR1), has a central role in the control of specific and shared phosphate starvation stress responses. In consonance with the importance of PHR1, we found that the PHR1-binding sequence, present in most PHR1 direct targets, is a crucial *cis* motif for Pi starvation responsiveness. An artificial promoter controlled by PHR1 recapitulates responsiveness to Pi starvation and to modulators of this response, qualifying PHR1 family members as central integrators in Pi starvation signalling. This central integrator system also controls most transcriptional repression responses to Pi starvation, indicating that they are an integral part of the adaptive response, and not a consequence of plant malfunction due to stress.

## Introduction

Plants have evolved adaptive responses to cope with growth under a variety of stress conditions. These responses involve changes that are specific to particular types of stress or shared by different stress types. A specific response to phosphate (Pi) starvation, for example, is increased Pi uptake capacity from the soil, whereas the induction of anthocyanin accumulation and acceleration of senescence are shared responses to many different kinds of stress [1]–[3]. In line with the overlap among physiological and developmental responses to different stress types, transcriptional responses overlap as well [4], [5]. An important question regarding transcriptional responses to stress is how specific and shared responses are regulated - are they controlled by the same regulatory systems or are there generic stress response regulators? A second question is the biological significance of transcriptional repression in stress responses. Is it mostly an integral part of the adaptive system or is it mainly an indirect consequence of plant malfunction due to stress?

We addressed these two questions, focussing on the Pi starvation stress response as a model in Arabidopsis. The importance of transcriptional control in the regulation of Pi starvation responses has already been established. The expression of a large number of genes is altered in response to Pi starvation (between 900 and 3000, depending on the study) [6]–[11]. The transcription factor (TF) PHR1 is a key regulatory component of Pi starvation responses in Arabidopsis [12]; PHR1 binds to an imperfect palindromic motif present in the promoters of many Pi starvation-induced genes. Loss of function mutation of PHR1 affects several Pi starvation responses, including alteration of root to shoot growth ratio, anthocyanin accumulation, and the expression of several Pi starvation-induced genes. Nonetheless, the extent of the role of PHR1 in Pi starvation responses has yet to be established. *PHR1* is part of a family of 15 genes in Arabidopsis (*MYB-CC* family). Some functional redundancy among family members has been suggested, based on the fact that the *phr1* effect on some Pi starvation-responsive genes is only partial [12].

In addition to PHR1, members of bHLH, WRKY, Zinc finger and R2R3 MYB families of TF are involved in the control of Pi starvation responses, although their exact positions in the signalling pathway have not been established [13]–[17]. Whereas *PHR1* is weakly transcriptionally responsive to Pi starvation, these other TF genes are highly responsive to Pi stress, suggesting that they act downstream of *PHR1*.

Additional mechanisms other than TF operate to regulate Pi starvation signalling. These include sumoylation [18], degradation by the proteosome, which probably involves the E2 ubiquitin conjugase-related enzyme (PHO2) [19]–[21], and control of Pi uptake efficiency via a phosphate transporter traffic facilitator (PHF1) [22], as well as several miRNA and antagonists (*IPS1* and related genes) of miRNA MiR399, which controls PHO2 activity [20], [23]–[27]. There is also a Pi starvation-induced gene family that encodes nuclear SPX domain-containing proteins, which affects responsiveness of several Pi starvation-induced genes through an unknown mechanism [28], [29]. *MiR399*, *IPS1* and *PHF1* are all under the control of PHR1, which itself is sumoylated *in vitro* by SIZ1, further strengthening the central role of PHR1 in the control of Pi starvation responses [12], [18], [20], [22].

Here we performed a physiological and transcriptomic analysis of Pi starvation responses in plants with altered PHR1(-like) activity, comparing mutants of *phr1*, *phr1-like1 (phl1)* and *phr1 phl1*, and *PHR1*-overexpressing transgenic lines. Results showed that PHR1 and PHL1 are partially redundant and have a central role in the control of physiological and molecular responses to Pi starvation, independent of whether these responses are specific to Pi starvation stress. They also indicate that a large proportion of the transcriptional repression responses to Pi starvation are part of the adaptive response to this stress, and that their control by PHR1(-like) is indirect. We also show the importance of the PHR1 binding sequence (P1BS) as an integrating *cis*-regulatory motif associated with genes that are highly induced by Pi starvation.

## Results

### Functional redundancy between PHR1 and PHR1-LIKE1 (PHL1) and their central role in the control of Pi starvation responses

**Figure 1:**
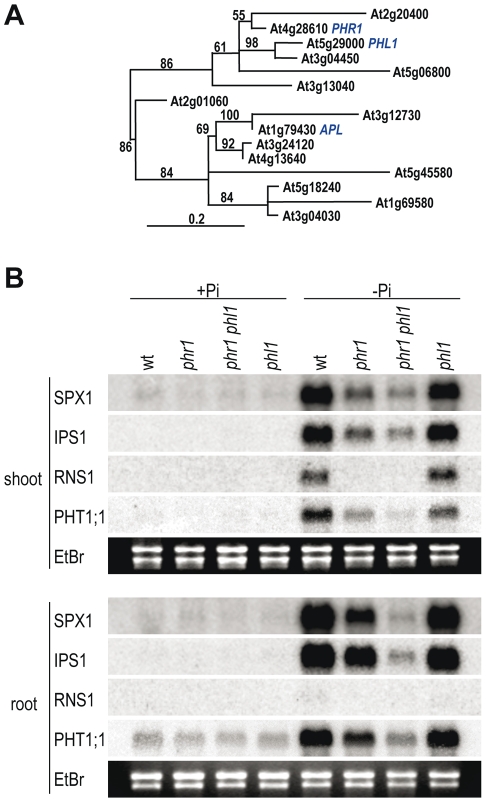
Functional redundancy of *PHR1* and *PHL1* in Pi starvation responsiveness of gene expression. (A) Phylogram of MYB-CC proteins from Arabidopsis, constructed using the Phylogeny-fr platform (www.phylogeny.fr) [70]. In addition to the AGI number, names are given for the functionally characterized members: PHOSPHATE STARVATION RESPONSE REGULATOR 1 (PHR1) [12]; PHR1-LIKE1 (PHL1; this study) ALTERED PHLOEM DEVELOPMENT (APL) [71]. The bootstrap value of each node is indicated (100 samples); nodes with bootstrap value <50 were collapsed. Only the conserved MYB and coiled-coil domains were used for alignment (Figure S1) and tree construction. (B) Northern analysis of the effect of *phr1* and *phl1* mutations on the expression of Pi starvation-responsive marker genes. Plants were grown for 7 days in Pi-rich or -lacking medium; RNA from roots and shoots was isolated separately and blots were hybridised sequentially to the probes *PHT1;1*, *RNS1*, *IPS1*, and *SPX1*. Ethidium bromide-stained rRNA was used as loading control.

*PHR1* mutants show distinct degrees of impairment of different Pi starvation responses, as evident in expression analyses of a set of Pi starvation-responsive genes [12]. Incomplete impairment of these responses could reflect partial gene redundancy, as PHR1 belongs to a transcription factor family with 15 close members (Figure 1A and Figure S1). It is also possible that more than a single regulatory system controls Pi starvation responses. To study the relationships between these possibilities, we searched for T-DNA mutations at *PHR1*-related genes in public databases; the two phylogenetically most closely related Arabidopsis genes for which a mutant was available were *At5g29000* and *At5g06800*. We selected *At5g29000*, which we term *PHR1-LIKE1* (*PHL1*) for further analysis, as it displayed a higher degree of amino acid identity with PHR1, and the T-DNA mutation disrupted the coding region of *PHL1* mRNA (Figure S1 and Figure S2A). We examined whether expression of *PHR1* and *PHL1* overlapped. Northern analysis showed that *PHL1* expression overlapped with that of *PHR1* in both shoots and roots under any Pi regime (Figure S2B). This observation was confirmed by the large overlap in *PHR1* and *PHL1* expression at different developmental stages, according to GENEVESTIGATOR gene expression data (https://www.genevestigator.com) [30] (Figure S2C). After generating a homozygous double mutant *phr1 phl1*, functional redundancy between PHR1 and PHL1 was shown by northern analysis (Figure 1B). Whereas the effect of the *phl1* mutation on Pi starvation responsiveness was barely detectable, we observed a synergistic effect of *phr1* and *phl1* mutations for expression of all genes examined. To be noted is the limited effect of these mutations on expression of Pi starvation induced genes in plants grown under a high Pi regimen, as shown by northern analysis and also using quantitative reverse transcription PCR (Q-RT-PCR) (Figure 1B and Figure S3).

For comparative purposes, we produced transgenic plants with the *phr1* background, overexpressing PHR1 fused to the rat glucocorticoid receptor domain (GR:PHR1) to allow dexamethasone (DEX)-inducible control of its activity [31]. Northern analysis showed that three independent lines overexpressing the GR:PHR1 fusion had DEX-dependent PHR1 activity (Figure S4). The effect of GR:PHR1 overexpression on gene expression was detected even when plants were grown under Pi sufficient conditions (Figure S4). These results are in agreement with previous reports [32], [33], and indicate that PHR1 overexpression can override, at least to some extent, the negative regulatory control that occurs at much more limited PHR1 levels in wild type plants.

**Figure 2:**
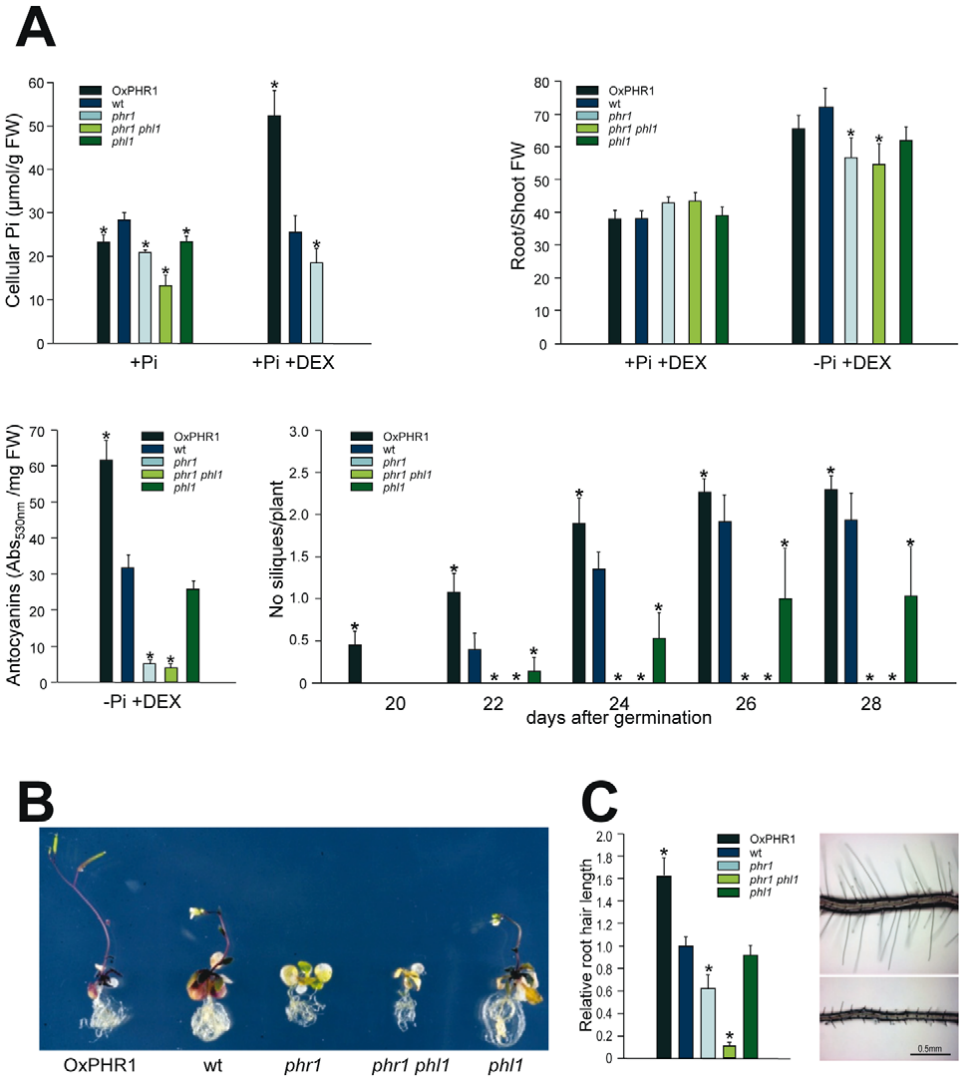
Effect of *phr1* and *phl1* mutations and PHR1 overexpression on physiological responses to Pi starvation. (A) Histograms of metabolic (Pi and anthocyanin content) and developmental (root/shoot fresh weight ratio and number of siliques per plant) parameters of wild type (wt), *phr1, phl1,* and *phr1 phl1* mutants, and GR:PHR1 overexpression line (OxPHR1). For analysis of Pi content, plants were grown for 12 days in complete medium (+Pi) and +Pi supplemented with 5 µM dexamethasone (+Pi+DEX). Anthocyanin content was measured in plants grown for 12 days in Pi-lacking medium supplemented with 5 µM DEX (−Pi+DEX). Root/shoot fresh weight (FW) ratio was measured in plants grown for 10 days in +Pi medium, and then transferred for 6 days to +Pi+DEX or −Pi+DEX media. Number of siliques was scored in plants grown for 9 days in +Pi and transferred to −Pi+DEX. Day 0 corresponds to start of germination. (B) Phenotypes of wild type, *phr1*, *phl1*, and *phr1 phl1* plants, and a OxPHR1 line grown for 9 days in complete medium, then transferred to −Pi+DEX for 13 days. The image reflects a phenotype frequent at the time examined. (C) Root hair size of wild type, *phr1*, *phl1*, and *phr1 phl1* plants, and a OxPHR1 line (left) and a detail (right) showing root hairs of wild type (top) and *phr1 phl1* plants (bottom). Plants were grown in vertical plates for 12 days in Pi-lacking medium. Scale bar, 0.5 mm. Asterisks indicate significant differences with wild type (p<0.05, Student's *t*-test).

Physiological tests were performed on wild type, *phr1*, *phl1* and *phr1 phl1* mutants, and transgenic plants overexpressing GR:PHR1 (Figure 2). In accordance with previous results [12], Pi accumulation in plants grown under Pi sufficient conditions was reduced in the *phr1* mutant (compared to wild type plants; Figure 2A). The *phl1* mutant had slightly, but significantly reduced Pi levels, and a further decrease in Pi accumulation was observed in the *phr1 phl1* double mutant, indicating partial functional redundancy between these two *MYB-CC* family genes. Conversely, Pi accumulation in DEX-treated GR:PHR1-overexpressing plants (*OxGR:PHR1*) was greatly increased with respect to that in wild type plants (Figure 2A). After Pi starvation, anthocyanins accumulate in leaves and stems of wild type plants; much less anthocyanin accumulated in the *phr1* mutant (Figure 2A). The effect on anthocyanin accumulation was negligible for *phl1*, however, and did not differ significantly between *phr1* and the *phr1 phl1* double mutant (Figure 2A). In contrast, anthocyanin accumulation was enhanced in *OxGR:PHR1* plants. Following Pi starvation, wild type plants show an increase in root to shoot growth ratio; this increase was significantly reduced in *phr1* mutants, whereas the *phl1* mutation had a negligible effect alone or in combination with *phr1*. In DEX-treated *phr1* GR:PHR1-overexpressing plants, the root to shoot growth ratio was similar to that of wild type plants in Pi starvation conditions (Figure 2A).

The effect of *phl1*, and in particular, of *phr1* and *phr1 phl1* mutations on senescence and silique formation was evident on plants grown in Pi starvation conditions, as these plants died before flowering (Figure 2A and 2B). In contrast, DEX-treated *OxGR:PHR1* plants showed slightly accelerated flowering and higher silique production. These findings concur with the idea that cell death in the mutants reflects a lack of correct protection against the stress inherent in Pi starvation, and that increased PHR1 activity results in increased reproductive success in these stress conditions.

The effect on root hair length was quite obvious when plants where grown in Pi-lacking medium in vertical plates; the *phr1* mutation affected root hair length, which was enhanced when combined with the *phl1* mutation (Figure 2C).

### PHL1 and PHR1 have similar *in vitro* DNA-binding properties

**Figure 3:**
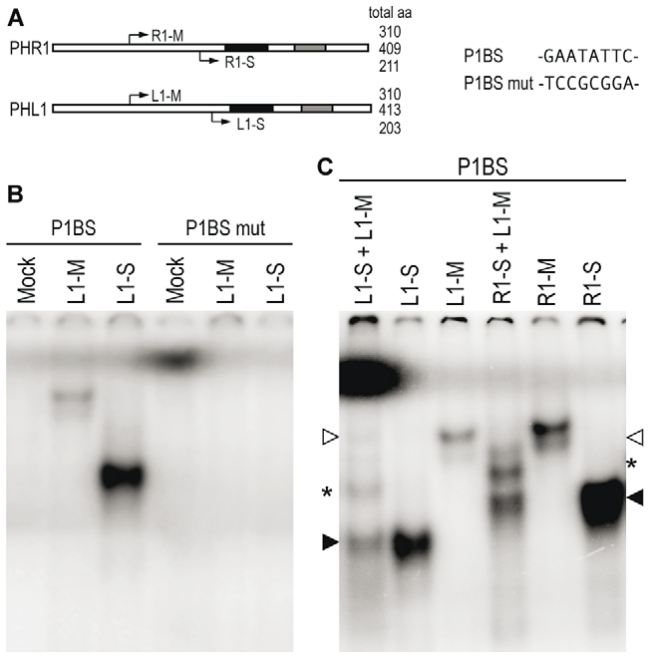
DNA-binding and dimerisation properties of PHL1. (A) Diagram showing the PHR1 and PHL1 proteins, including MYB (black) and the predicted coiled-coil domains (grey), indicating the start of the N-terminally truncated versions of PHR1 (medium-length PHR1, R1-M; short PHR1, R1-S) and PHL1 (medium-length PHL1, L1-M; short PHL1, L1-S) used (left). The core sequence of the oligonucleotide containing the PHR1 binding sequence (P1BS) and the mutated version (P1BS mut) are shown (right). (B) EMSA showing binding to P1BS, but not to its mutant version, of N-terminal forms of PHL1. (C) PHL1 dimerisation determined by EMSA with the two N-terminally truncated versions of PHL1 and PHR1. Proteins were translated *in vitro* alone or in combination. Arrows show the position of the homodimers formed with short (closed arrows) and medium-length proteins (open arrows); asterisks show the position of homo- or heterodimers formed by the combination of short and medium-length proteins. A mock translation mixture (Mock) was used as control.

Given the partial functional redundancy between PHR1 and PHL1, as shown by our analyses of *phr1* and *phl1* single and double mutants, we examined whether these two proteins had similar DNA binding properties and whether they were able to heterodimerise. For these studies, we used two N-terminally truncated versions of each protein obtained by *in vitro* translation, since a previous study with PHR1 showed that *in vitro*-translated N-terminally truncated PHR1 protein had similar DNA binding specificity but higher affinity than the full length protein [12]. The two deletions removed 99 or 198 N-terminal amino acids of PHR1 and 103 or 210 residues of PHL1 (Figure 3A). Electrophoretic mobility shift assays (EMSA) indicated that both PHL1 versions could interact with P1BS sequences, the prototype PHR1 binding site (Figure 3B). EMSA with the two cotranslated truncated PHL1 versions showed the appearance of a band of intermediate mobility, in addition to those corresponding to the medium and short versions of truncated PHL1; this was indicative of the self-dimerisation properties of PHL1 [34], as also observed for PHR1 [12]. Intermediate mobility bands were also observed when the medium size PHL1 version was cotranslated with the short PHR1 version, indicating that they can form heterodimers (Figure 3C).

The ability of PHL1 and PHR1 to heterodimerise was confirmed by identification of PHL1 as a PHR1-interacting protein in a yeast two-hybrid assay (Figure S5).

### Broad effects of *phr1* and *phr1 phl1* mutants on transcriptional responsiveness to Pi starvation

To examine the correspondence between the effects of *phr1* and combined *phr1 phl1* mutations on physiological responses and transcriptional responses to Pi starvation, we performed transcriptomic analysis in wild type, as well as single *phr1* and double *phr1 phl1* mutants. For these assays, wild type plants were germinated and grown for 7 days in Pi-sufficient and -starvation conditions, and mutant plants were grown in Pi starvation conditions. The use of long-term stress treatment for the analysis was aimed to identify the long-term effects of these mutations.

For transcriptomic analyses, we collected RNA separately from shoots and roots in three independent replicates obtained over a two-month interval. A total of 1873 and 704 genes were upregulated, and 1795 and 326 downregulated in Pi-starved shoots and roots, respectively (cut-off values 2-fold, false discovery rate (FDR)<0.05; Table 1 and Table S1). The effect of the *phr1* mutation, particularly when combined with *phl1*, on the expression of these Pi starvation-responsive genes was striking (Table 1). Of the genes whose expression was induced two-fold or more in the wild type plants in response to Pi starvation, 68% and 47% showed at least two-fold reduced expression in the shoots and roots, respectively, of the Pi-starved *phr1 phl1* double mutant compared to wild type in the same conditions. In contrast, only 1.4% and 2% of Pi starvation-induced genes in shoots and roots showed increased expression in the Pi-starved double mutant. These numbers are even more extreme if only genes induced four-fold or more in wild type plants are analysed, or if the cut-off values in the mutant *versus* wild type comparison are relaxed (1.5-fold, FDR<0.1) (Table 1). For example, >80% and 60% of the Pi starvation-induced genes in shoots and roots, respectively, show reduced expression in the Pi-starved *phr1 phl1* double mutant using cut-off values of 1.5-fold, FDR<0.1. The situation is similar for the repressed genes, as 70% and 46% of genes repressed in shoot and roots, respectively, of wild type plants grown in Pi starvation conditions showed higher expression in the Pi-starved double mutant. These data underline the central regulatory role of *PHR1(-like)* genes in the transcriptional control of Pi starvation responses. Reciprocally, *phr1* and *phl1* mutations mostly affect expression of Pi responsive genes (Figure S6). For example, Pi starved shoots of the double mutant display reduced expression relative to wild type levels of almost 90% of highly (>4×) Pi starvation induced genes, while this proportion falls below 2% for non Pi starvation responsive (or Pi starvation repressed) genes (Figure S6).

**Table 1:** Transcriptomic responses to Pi starvation and effect of *phr1* and *phl1* mutations.

			Reduced expression in Pi-starved mutants *vs.* wt				Increased expression in Pi-starved mutants *vs.* wt			
			*phr1*		*phr1 phl1*		*phr1*		*phr1 phl1*	
wt	FDR<0.05	No of genes	2× FDR<0.05	1.5× FDR<0.1	2× FDR<0.05	1.5× FDR<0.1	2× FDR<0.05	1.5× FDR<0.1	2× FDR<0.05	1.5× FDR<0.1
**Shoot Up**	**2×**	**1873**	57.1	73.9	68.1	81.5	1.5	2.8	1.4	1.9
	**4×**	**656**	82.8	87.8	89.2	93.1	0.9	1.1	0.6	0.9
**Root Up**	**2×**	**704**	22.0	39.1	46.9	62.5	2.6	4.3	2.0	4.3
	**4×**	**184**	42.4	60.9	78.8	83.7	0.5	2.7	1.1	1.6

			Increased expression in Pi-starved mutants *vs.* wt				Reduced expression in Pi-starved mutants *vs.* wt			
			*phr1*		*phr1-phl1*		*phr1*		*phr1-phl1*	
wt	FDR<0.05	No of genes	2× FDR<0.05	1.5× FDR<0.1	2× FDR<0.05	1.5× FDR<0.1	2× FDR<0.05	1.5× FDR<0.1	2× FDR<0.05	1.5× FDR<0.1
**Shoot Down**	**2×**	**1795**	41.7	60.2	49.9	69.5	0.8	1.4	0.8	1.2
	**4×**	**215**	61.9	73.0	69.3	80.5	0.0	0.0	0.0	0.0
**Root Down**	**2×**	**326**	25.2	34.4	37.1	46.0	1.5	3.1	2.5	5.5
	**4×**	**65**	49.2	53.8	47.7	52.3	3.1	9.2	6.2	9.2

Total number of Pi starvation-induced (Up) and -repressed (Down) genes in wild type shoots and roots. The percentage is indicated of these Pi starvation-responsive genes that show reduced or increased expression in Pi-starved single *phr1* and double *phr1 phl1* mutants. Analysis was done using two different cut-off values for Pi starvation-responsive genes in wild type (two-fold (2×) and four-fold (4×), FDR<0.05) and in mutants (2×, FDR<0.05; 1.5-fold (1.5×), FDR<0.1).

To measure the extent of functional redundancy between *PHR1* and *PHL1*, we examined the Pi starvation-responsive genes whose expression was greatly altered in the double mutant compared to *phr1*. Only a small proportion of Pi starvation-responsive genes showed more than two-fold altered expression in the *phr1 phl1* double mutant compared to *phr1* (200 Pi starvation-induced and 82 Pi starvation-repressed genes displayed more than two-fold reduced and increased expression, respectively, in *phr1 phl1 vs. phr1*; Table S2). Of the genes showing altered expression in *phr1 phl1* compared to *phr1*, only 30% did not show altered expression in Pi-starved single *phr1* mutant *vs.* Pi-starved wild type plants, indicating a large functional overlap between *phr1* and *phl1*.

We used MAPMAN ontology tools to obtain an overview of Pi starvation-responsive genes involved in metabolism and regulation (http://mapman.gabipd.org/web/guest/home) [35] (Figure S7). Pi starvation had a broad effect on genes involved in all aspects of metabolism. In particular, induced genes were greatly enriched in secondary metabolism biosynthetic genes, especially those of phenylpropanoids (Figure S7A). There was also an increase in biosynthetic genes of sulpholipids and galactolipids, which replace phospholipids under Pi-limiting conditions as previously reported [36]–[38], and of tetrapyrroles. Pi starvation also had a large effect on transcriptional repression of genes involved in light reactions of photosynthesis and in photorespiration (Figure S7A). These are likely protective responses as they would reduce the potential generation of reactive oxygen species. Pi starvation-triggered changes in the transcription of regulatory components showed notable effects on genes encoding transcription factors, components of protein degradation machinery, hormone biosynthesis and signalling, and calcium-based regulation, with induction more prominent than repression (Figure S7B). Our findings are qualitatively similar to those in previous reports [9], [10].

We compared our set of Pi starvation-induced and -repressed genes with the sets of genes responsive to different types of stress or hormones available at the GENEVESTIGATOR database [30] (Table S3). In most cases, there were significant overlaps between the set of genes responsive to Pi starvation and those responsive to other types of stress and, as reported, there were also many significant overlaps with hormone-responsive gene sets [39]. To infer whether control of shared genes, i.e., responsive to Pi starvation and other stresses, could occur through independent regulatory systems (involving different stress type-specific TF) or, alternatively, could in part use common regulatory components, we examined the representation of TF genes in the sets of shared genes that respond to Pi starvation and to other stress types. In most cases, we found that TF genes were equally over-represented relative to non-TF genes in the sets of shared genes (Table S3); this favours the idea that transcriptional control of genes that respond to two stress types in part uses common regulatory components. Two exceptions corresponded to hydrogen peroxide treatment and low nitrate growth conditions, whose induced genes are significantly enriched in Pi starvation-induced genes; enrichment was much weaker or non-existent for Pi starvation-induced TF, however, raising the possibility that in these cases, part of the shared response is controlled by independent TF.

Finally, we studied the effect of Pi starvation on general stress response (GSR) genes. Two independent studies recently identified sets of general stress-induced genes [4], [5]. There is considerable overrepresentation of these genes in our set of Pi starvation genes induced two-fold or more (>44% *vs.* a predicted 9%) (Table 2). A large proportion of these general stress-induced genes responsive to Pi starvation show reduced expression in the Pi-starved *phr1 phl1* double mutant (∼70%), indicating that general stress responses associated to Pi starvation are controlled by PHR1(-like) TF.

**Table 2:** Pi starvation responsiveness of general stress response (*GSR*) genes and their control by PHR1(-like).

Experiment	No GSR genes	PSI-GSR genes	% PSI-GSR genes affected in *phr1 phl1*
**Ma and Bohnert (2007)**	277	127	67.2
**Walley ** ***et al.*** ** (2007)**	161	069	75.4

The number of *GSR* genes reported in two previous studies [4], [5], as well as the number of *GSR* genes induced by Pi starvation (PSI-GSR; cut-off value 2×, FDR<0.05) is given. The number is also shown of Pi starvation-induced *GSR* genes that display lower expression after Pi starvation in *phr1 phl1* double mutant *versus* wild type plants (cut-off value 1.5×, FDR<0.1).

### Direct targets of PHR1 are greatly enriched in P1BS-containing Pi starvation-induced genes

To examine direct targets of PHR1, we followed the strategy originally described by Galaktionov and Sablowski [40], [41], which is based on the use of a transgenic *phr1* mutant plants expressing the GR:PHR1 fusion (*OxGR:PHR1 phr1*), whose activity is postranslationally controlled by DEX. Gene expression analysis following DEX-mediated PHR1 activation and the concomitant inhibition of translation with cycloheximide (CHX), which prevents PHR1 effects on the expression of secondary targets, will inform on PHR1 direct targets. For this study, *OxGR:PHR1 phr1* and *phr1* plants were grown in complete (+Pi) liquid medium for 7 days, then transferred for 2 days to phosphate-lacking (−Pi) medium. Plants were then supplemented with 5 µM DEX and 10 µM CHX, and incubated for 6 h before harvest. Total RNA was isolated from 3 independent samples of *OxGR:PHR1 phr1* and *phr1* plants and transcriptomic analysis was performed. Using standard cut-off values (two-fold, FDR<0.05), 319 and 21 genes showed increased or decreased expression in *OxGR:PHR1 phr1 vs. phr1* mutant plants, respectively. A considerable overlap was found between the set of genes with increased expression in CHX-treated *OxGR:PHR1 phr1* plants with the set of Pi starvation-induced genes (210 out of 319), whereas there was almost no overlap between genes with reduced expression in CHX-treated *OxGR:PHR1 phr1* plants and Pi starvation-repressed genes (1 gene; Table 3 and Table S4). This finding indicates that PHR1 is a *bona fide* transcriptional activator and that PHR1 control of Pi starvation-repressed genes is indirect.

**Table 3:** PHR1 direct targets.

*OxGR:PHR1phr1 vs. phr1*		−Pi Up regulated genes	−Pi Down regulated genes
***Up***	**319**	210	4
**Down**	**021**	007	1

The total number of genes with higher (Up) or lower (Down) expression in the *phr1* mutant overexpressing the GR:PHR1 fusion compared to *phr1* mutant plants is shown. The number is shown of coincidences with Pi starvation up- or downregulated genes. In this experiment, plants were grown for 7 days in +Pi medium, transferred for 2 days to −Pi medium, and then treated with 5 µM DEX and 10 µM CHX for 6 h before harvest.

**Figure 4:**
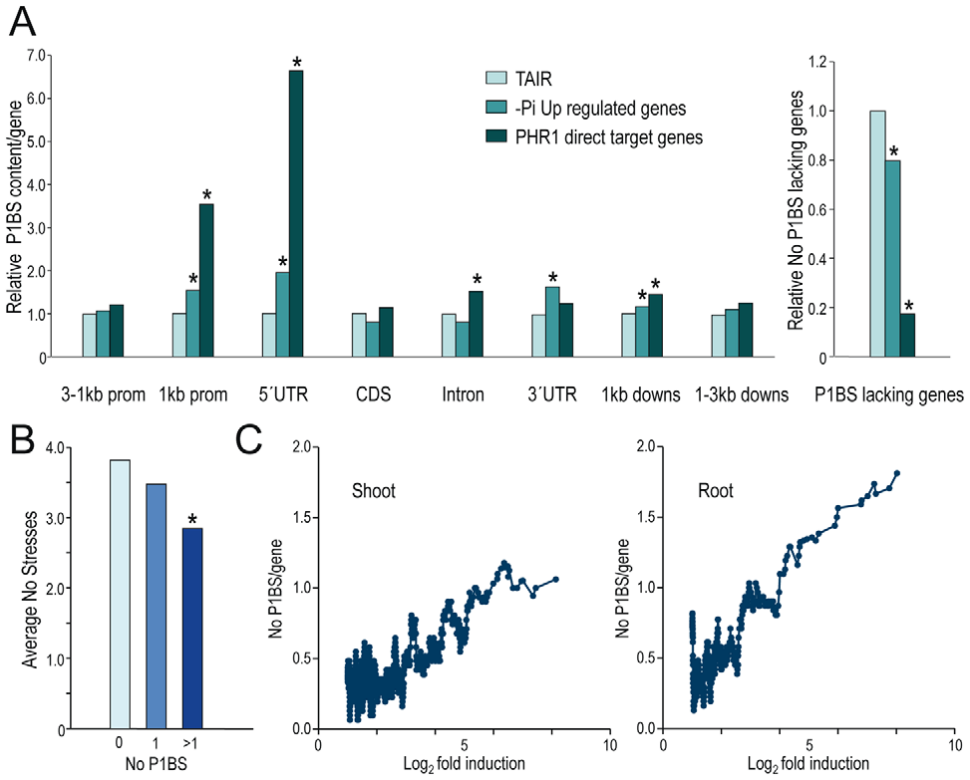
P1BS distribution over different gene parts of PHR1 direct targets and Pi starvation-responsive genes. (A) P1BS content per gene in different gene parts (distal promoter region, 3-1 kb prom; proximal promoter region, 1kb prom; 5′UTR, coding region, CDS; Intron; 3′UTR; proximal downstream region, 1 kb downs; distal downstream region, 1–3 kb downs) (left) and proportion of genes lacking P1BS in any of these gene parts (right). The P1BS content of the average Arabidopsis genes, represented in the Affimetrix chip used in transcriptomic analyses, is taken arbitrarily as 1. (B) Average number of other stresses in which Pi starvation-induced genes are also induced relative to the number of P1BS motifs in the 1 kb proximal promoter region. Data for induction by other stress types were obtained from 28 stress conditions for which transcriptomic data were available in the GENEVESTIGATOR database (https://www.genevestigator.com) [30]. Asterisks in A and B represent significant differences (p<0.01 using the χ^2^ test). (C) Relation between the number of P1BS motifs in the 1 kb promoter proximal region and log_2_ *x*-fold induction. The number of P1BS/gene (No P1BS/gene) was calculated as the average content of P1BS motifs over successive sets of 30 genes, measured at a one-gene interval, ordered according to inducibility by Pi starvation.

To substantiate the conclusion that PHR1 control of Pi starvation-repressed genes is indirect, we tested for P1BS in different parts of the Pi starvation-responsive genes and in PHR1 direct targets. Direct targets were enriched in P1BS sequences in all parts of the gene compared to average Arabidopsis genes. As a result, only 3% of PHR1 direct targets did not have a P1BS site in the region encompassing 3 kb of the promoter region to 3 kb downstream, compared to 17% for average Arabidopsis genes. Enrichment was especially high in the 1 kb proximal promoter region and even higher in the 5′UTR. Although the 3′UTR of direct targets was only weakly enriched in P1BS sequences, P1BS was significantly enriched in the 3′UTR of the whole set of Pi starvation-induced genes (Figure 4 and Table S5).

We next tested whether specificity of Pi starvation inducibility correlated with P1BS content in the promoter. We examined the average number of other stresses in which Pi starvation-induced genes are also induced relative to the presence of none, one, or more than one P1BS in the 1 kb proximal promoter region, 5′UTR, 3′UTR, introns or 1 kb proximal downstream region, or in any combination of these, in which the set of Pi starvation-induced genes and/or PHR1 direct targets showed a significantly higher P1BS levels compared to average Arabidopsis genes (Figure 4A and Table S5). P1BS content in the proximal promoter region, the 5′UTR or introns was associated with slightly higher specificity of Pi starvation-responsiveness; however, the difference in specificity was insufficient to ascribe specificity to the class of genes containing P1BS (Figure 4B and Figure S8A).

We examined whether genes with P1BS in their promoters, 5′UTR, 3′UTR, introns and the 1 kb downstream region were induced at a higher level by Pi starvation. Analysis of P1BS representation relative to the x-fold induction showed a striking correlation between inducibility and P1BS content in the 1 kb proximal promoter region, whereas P1BS content in other gene regions showed no correlation with inducibility (Figure 4C and Figure S8B).

### The P1BS sequence is a key *cis*-regulatory motif in Pi starvation signalling

To confirm the importance of P1BS as key *cis*-regulatory motifs in Pi starvation signalling, we performed two types of experiments: i) evaluation of the effect of P1BS mutation on Pi starvation-responsive genes and ii) analysis of Pi starvation responsiveness mediated by a minimal promoter containing multimerised P1BS.

**Figure 5:**
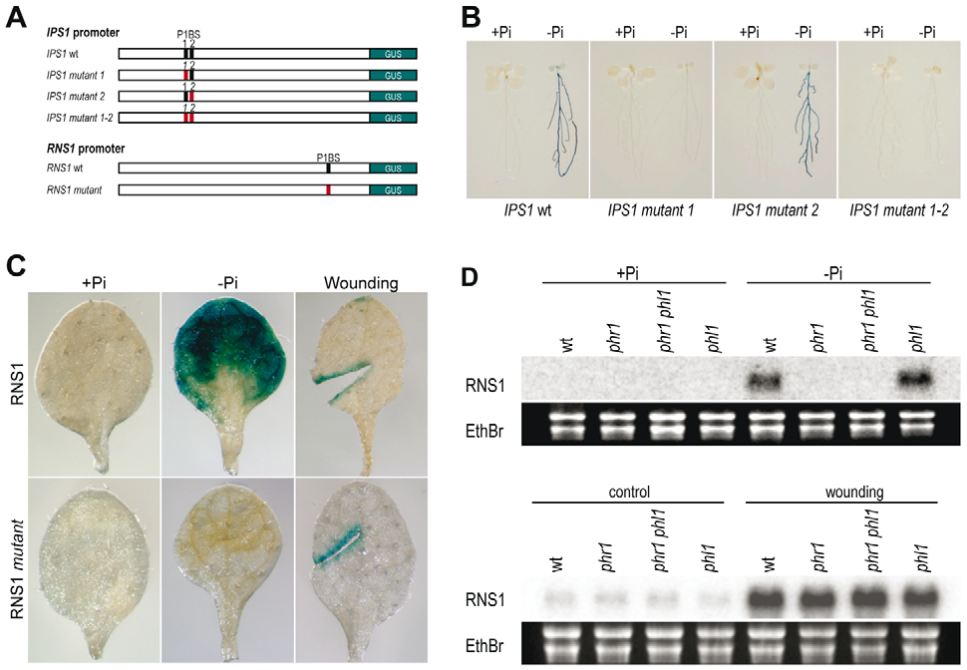
P1BS is a key *cis*-regulatory motif in Pi starvation responsiveness. (A) Diagram shows *IPS1:GUS* and *RNS1:GUS* reporter genes and mutated versions thereof. The P1BS motifs in each gene are highlighted with a vertical bar (wild type, black; mutant, red). (B,C) Histochemical analysis of GUS activity driven by wild type and mutated versions of the *IPS1:GUS* reporter gene in plants grown in +Pi or −Pi media (B), or driven by wild-type and a mutated version of the *RNS1:GUS* reporter gene in plants grown in +Pi and +Pi media after wounding (C). (D) Northern analysis of *RNS1* gene expression in wild type and *phr1*, *phl1*, and *phr1 phl1* mutants in response to Pi starvation or wounding. Ethidium bromide-stained rRNA was used as loading control. For the Pi starvation experiment, plants were grown for 7 days in +Pi or −Pi media and RNA prepared from shoots. For the wounding experiment, plants were grown for 14 days, and wounded and unwounded control leaves were harvested for histochemical analysis of GUS activity and for RNA isolation 8 h after wounding.

For the first experiment, we selected the promoters of two genes, *IPS1*, a highly specific Pi starvation-induced gene [42], and *RNS1*, which is also responsive to wounding stress [43]. A 1 kb DNA fragment containing the promoter proximal region up to the first initiation codon in the transcribed region was obtained for each gene by PCR amplification of genomic DNA. We also prepared mutants in which the P1BS sites of each gene were impaired. For the *IPS1* promoter, which has two P1BS, we obtained single mutants of each P1BS and a double mutant of both. Transgenic plants harbouring these promoters or their mutant versions fused to the coding region of *GUS* in the pBI101 binary vector [44] were obtained (Figure 5A).

In the case of wild type *IPS1* constructs (*IPS1:GUS*), 10 of 10 transgenic plants examined showed Pi starvation-induced GUS activity. Mutation of P1BS-2 had no effect on Pi starvation responsiveness (9 of 10 transgenic plants showed Pi starvation-induced GUS activity), whereas mutation of P1BS-1 abolished Pi starvation responsiveness (10 of 10 plants had no GUS activity; see example in Figure 5B). For wild type *RNS1*, 9 of 10 transgenic plants displayed Pi starvation-induced GUS activity, whereas P1BS impairment resulted in no Pi starvation-induced GUS activity (Figure 5C). In the case of *RNS1*, we also examined responsiveness to wounding. Both the wild type *RNS1* promoter and the mutant promoter impaired in P1BS showed similar wounding-induced GUS activity (Figure 5C). We examined whether *phr1* and/or *phl1* mutations affected *RNS1* expression. Northern analysis indicated that mutation of *PHR1* and *PHL1*, while impairing Pi starvation responsiveness, had no effect on the *RNS1* wounding response (Figure 5D). These results point to a critical role for P1BS in Pi starvation responsiveness and, in the context of non-specific Pi starvation-responsive genes, indicate that PHR1(-like) and P1BS are not necessarily required for responsiveness to stresses other than Pi starvation. In addition, it is evident from the case of *IPS1* that not all P1BS motifs in a promoter are equally relevant for Pi starvation responsiveness. Other architectural determinants such as nucleosome positioning and P1BS organisation with respect to additional *cis* motifs might determine P1BS function.

**Figure 6:**
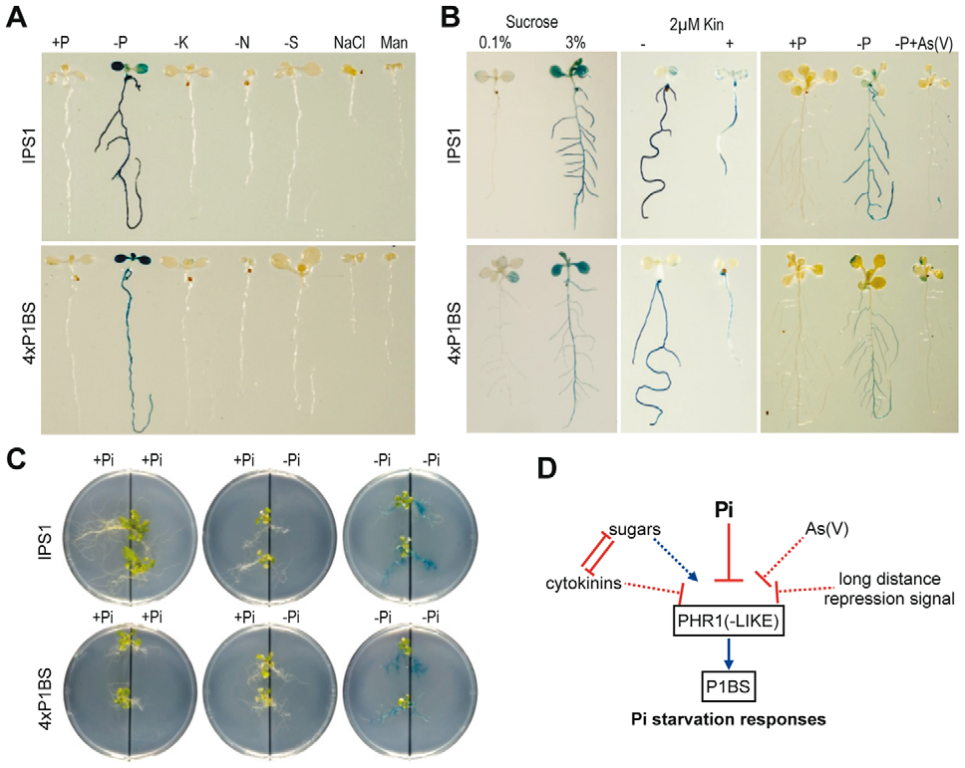
P1BS is an integrator *cis* motif in Pi starvation signalling. (A–C) Histochemical analysis of GUS activity driven by *IPS1:GUS* (IPS1) and *4xP1BS:GUS*, a reporter gene containing a synthetic promoter harbouring four tandem copies of P1BS fused to the −46 minimal 35S promoter from CaMV (4xP1BS). (A) Response of reporter genes to different types of nutrient starvation stress and to salt and osmotic stress. Plants were grown for 7 days in +Pi or −Pi medium, or media lacking potassium (−K), nitrogen (−N) or sulphur (−S). The effect of saline and osmotic stress was analysed in plants grown for 7 days in complete media supplemented with 150 mM NaCl (NaCl) or 300 mM mannitol (Man), respectively. (B) Response of reporter genes to known agonists (sucrose) or antagonists (cytokinins, arsenate) of the Pi starvation response. To analyse the effect of sucrose, plants were grown for 7 days in +Pi medium in low sucrose (0.1%) and transferred for 3 days to −Pi medium containing two concentrations of sucrose (0.1% or 3%; left). To examine the cytokinin effect, plants were grown for 5 days in −Pi medium, alone or with 2 µM kinetin (Kin; centre). Plants (right) were grown for 7 days in complete liquid medium and transferred for 4 days to +Pi or −Pi media alone or with 30 µM arsenate [As(V)]. (C) Response of reporter genes to long distance repression in a split root assay. Plants were grown for 7 days in complete medium, for 4 additional days on −Pi medium, then transferred for 4 days to split plates with compartments containing +Pi or −Pi media as indicated. (D) Model showing the integrator role of P1BS and consequently PHR1(-like) in Pi starvation signalling.

To analyse the capacity of P1BS to mediate Pi starvation responsiveness, we fused four tandem copies of P1BS to the −46 minimal 35S promoter from CaMV (*4xP1BS:GUS*) [45]. Transgenic plants harbouring this construct were fully responsive to Pi starvation (9 of 10 independent lines). We chose one of these lines to study the specificity of Pi starvation responsiveness and the effect of known agonists (sucrose) [46]–[48] or antagonists (cytokinins and arsenate) [39], [46], [49]. As in the case of *IPS1:GUS*, the *4xP1BS:GUS* construct was highly responsive to Pi starvation, but not to other types of stress (nitrogen, potassium and sulphur starvation, and salt and osmotic stress); in addition, it was responsive to the stimulatory effects of sucrose and the repressing effect of arsenate and cytokinins (Figure 6A and 6B).

Systemic repression is a characteristic type of control in nutrient physiology; it stands for the fact that most responses to nutritional deficiency are determined by shoot nutritional status rather than by the local nutrient concentration in the vicinity of the root system [50]. To evaluate whether systemic repression is signalled through P1BS, we used a split root assay in which part of the root system of Pi-starved plants was placed in Pi-lacking medium and the other part in Pi-rich medium. GUS activity was not detected in the Pi-lacking parts of the roots in the split root assay (Figure 6C). These results define P1BS and, consequently, PHR1(-like) TF as central integrators in Pi starvation signalling (Figure 6D).

### Phylogenetic footprinting shows combinatorial action of P1BS in Pi starvation responsiveness of wild type promoters

**Figure 7:**
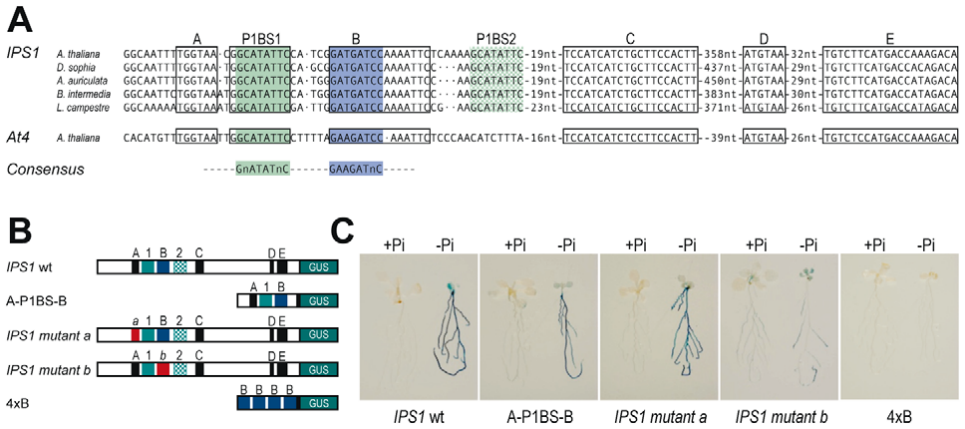
Phylogenetic footprinting and mutational analysis of the *IPS1* promoter. (A) Alignment of the promoter region of *IPS1* from five *Brassicaceae* species (*Arabidopsis thaliana*, *Descurainia sophia*, *Brassica intermedia*, *Arabis auriculata* and *Lepidium campestre*), which is conserved in the highly related *At4* gene. A consensus sequence is shown indicating conservation of P1BS closely linked to motif B in the promoter of more distant *IPS1-related* genes from Arabidopsis and from other species (*Medicago truncatula*, *Solanum lycopersicum*, *Populus trichocarpa* and *Zea mays*). Conserved regions between *IPS1* orthologs and *At4* are boxed; the P1BS1 and B motifs conserved in distantly related *IPS1* family members are shown in green and blue, respectively. The P1BS2 motif conserved only among *IPS1* orthologues is highlighted (pale green). (B) Diagram shows different *IPS1* promoter-derived reporter constructs: wild type 1 kb *IPS1* promoter region (IPS1 wt), including P1BS (green boxes), motif B (blue box) and motifs A, C, D and E (black boxes); a 42-bp *IPS1* promoter fragment (A-P1BS-B), including motifs A, P1BS1 and B; four tandem copies of motif B (4xB). Both A-P1BS-B and 4xB were fused to the −46 minimal promoter from the CaMV 35S gene fused to the coding region of the *GUS* reporter; versions of the *IPS1* promoter with either motif A or motif B mutated (red boxes) were fused to the coding region of the *GUS* reporter. (C) Histochemical analysis of GUS activity driven by wild type *IPS1:GUS* and derived reporter constructs. Plants were grown for 7 days in +Pi or −Pi media.

To examine whether P1BS sequences are sufficient in the context of a natural promoter to mediate Pi starvation responsiveness, we performed phylogenetic footprinting analysis to search for conserved *cis*-regulatory regions that could be relevant in the control of gene expression. For this analysis, we examined the promoter of the highly specific Pi starvation- responsive *IPS1* gene. Using oligo-adapted PCR amplification with a conserved region of *IPS1*, we amplified fragments containing the promoter region of orthologous genes from four different *Brassicaceae* species (Figure S9). Sequence alignment showed two highly conserved regions, spanning from nt −626 to −527 and from nt −280 to −109 from the first ATG in the transcribed region of *IPS1* (Figure S9). As this analysis did not provide sufficient resolution to identify *cis*-regulatory motifs, we included *At4* in the alignment, as it is also responsive to Pi starvation and is the most closely related *IPS1* homologue in Arabidopsis [25]; we thus delimited the candidates for *cis*-regulatory sequences to six short motifs (motifs A to E and P1BS1; Figure 7 and Figure S9). Further inspection of additional members of the family in Arabidopsis and other species such as tomato, medicago, maize and poplar showed that two of these six conserved motifs were also conserved outside the *Brassicaceae* family (P1BS1 and B motifs; Figure 7A). Fusion of the region encompassing motifs A-P1BS-B to the −46 minimal 35S promoter (*A-P1BS-B:GUS*) showed that this region is sufficient to mediate Pi starvation responsiveness of a GUS reporter gene (Figure 7B and 7C). Mutational analyses indicated that whereas impairment of motif A had no effect, mutation of motif B abolished Pi starvation responsiveness and resulted in weak constitutive expression of the mutant gene (Figure 7B and 7C). These results indicate that motif B acts in concert with P1BS to mediate Pi starvation responsiveness.

We also analysed whether motif B could drive Pi starvation inducibility, as is the case of P1BS. An artificial gene containing four tandem copies of motif B fused to the -46 minimal 35S promoter from CaMV and the *GUS* coding region (*4xB:GUS*) did not show any GUS activity, even in Pi starvation conditions (10 of 10 independent transgenic lines; Figure 7B and 7C).

To evaluate a possible informatic approach to predict the relevance of motif B and not of other conserved motifs in the context of Pi starvation responsiveness, we examined whether the conserved motifs A and B were overrepresented in the promoters of our set of Pi starvation-responsive genes. Neither of these conserved motifs were significantly overrepresented; nonetheless, we found a clear overrepresentation of motif B in combination with P1BS when the distance between the two motifs was restricted to 25 nt (26 observed *vs.* 12.4 predicted, p<0.0002; Table S6). These data, including the result using the artificial *4xB:GUS* gene, strongly suggest that the role of motif B in Pi starvation responsiveness is subsumed to that of P1BS, and that it is likely that PHR1(-like) proteins interact with a yet to be identified TF that interacts with motif B, directly or via a co-adaptor protein.

## Discussion

Plants rely on adaptive systems to protect themselves from different types of stress. One of these adaptive systems is that for Pi starvation stress, in which transcription factor PHR1 from the MYB-CC family plays an important regulatory role [12], although its extent remains unknown. Based on physiological and transcriptomic analysis of plants with altered expression of *PHR1* and of the closely related gene *PHL1*, in this study we i) show the central role of *PHR1* and functionally redundant *PHL1* in the control of Pi starvation responses, ii) identify PHR1 direct targets and the potential importance of pre-existing, shared regulatory components in PHR1-mediated indirect control, and iii) highlight the relevance of the PHR1 binding site (P1BS), in concert with other *cis*-regulatory motifs, in the direct control of Pi starvation-induced genes by PHR1. This type of regulatory architecture, involving a central integrator based on a single class of transcription factor, makes Pi starvation stress a suitable system in which to gain insights into general plant stress physiology, including the role of transcriptional repression in stress responses.

### Central role of functionally redundant *PHR1* and *PHL1* in the control of plant responses to Pi starvation

The partial functional redundancy between *PHR1* and *PHL1* is indicated by the additive or synergistic effects of mutations in these two genes on most of the traits examined, including transcriptional responsiveness to Pi starvation. Redundancy probably involves additional members of the *MYC-CC* family, since mutation of both *PHR1* and *PHL1* does not fully abolish Pi starvation responses. For instance, *IPS1* is still weakly responsive in the *phr1 phl1* double mutant, and we demonstrate that mutation of a P1BS site in *IPS1* completely abolishes the Pi starvation response of this promoter (Figure 1B and Figure 5B). In line with this partial functional redundancy, PHR1 and PHL1 have similar DNA binding specificity and can heterodimerise.

The use of plants with different PHR1(-like) activity levels (*phr1* and *phl1* single mutants, *phr1 phl1* double mutant, and PHR1-overexpressing plants) confirmed the essential role of *PHR1* and *PHL1* in the control of intracellular Pi concentrations and anthocyanin accumulation [12], as well as in other aspects of the response, such as root hair length, silique formation and senescence (Figure 2). The observed effect of *phr1* and *phl1* mutations on Pi levels of plants grown under a Pi rich regimen contrasts with the limited effect of these mutations on expression of Pi starvation induced genes in plants grown under these conditions. This could reflect a partial compensation of a lower amount of PHR1(-like) protein in these mutants with a higher activity of the remaining PHR1(-like) protein (likely encoded by PHR1-related genes), as the level of Pi, which inhibits PHR1(like), in mutants grown in +Pi medium is lower than in the wild type. The observed effect of the *phr1* mutation on root hair formation supports a previous finding in rice, in which overexpression of a rice PHR1 homologue was shown to affect root hair length and density [33]. Given that root hair response is dependent on local Pi concentration in the root surroundings rather that on shoot Pi concentration [51], our data indicate that *PHR1* also controls at least part of local Pi-dependent responses. The results also show the importance of a proper response to nutrient stress for reproductive success, which is enhanced in PHR1-overexpresssing plants (Figure 2).

Our transcriptomic analyses reveal the large quantitative dimension of the Pi starvation transcriptional response and the central regulatory role of PHR1 and PHL1. A total of 4170 genes, representing 18.5% of the genes analysed, displayed Pi starvation responsiveness (Table 1); of these, 75% of induced and 65% of repressed genes showed decreased and increased expression, respectively, in the Pi-starved *phr1 phl1* double mutant (Table 1), indicative of reduced responsiveness in the mutant lines. There is no precedent for a small number of related TF controlling a complex stress response to such a large extent, although a quantitatively similar role was described for two Snf1-related kinases, KIN10 and KIN11, that act as central integrators in sugar/energy depletion responses [52].

As for physiological and developmental responses, many Pi starvation-responsive genes are also responsive to other stresses, yet their responsiveness to Pi starvation is compromised in the *phr1* and *phr1 phl1* mutants. Non-specific molecular responses can thus be controlled by stress type-specific regulatory systems. A paradigmatic example of this is represented by general stress response (*GSR*) genes; these genes, identified in two independent studies, have been ascribed to an independent regulatory system [4], [5]. Nonetheless, a large proportion of Pi starvation-responsive *GSR* genes are controlled by PHR1(-like) (Table 2). One way to reconcile the existence of an independent regulatory system for *GSR* genes and the observation that they are controlled by PHR1(-like) TF is that PHR1(-like) TF exert their regulatory role on these genes by acting on the GSR regulatory system.

Also noteworthy is the finding that 65% of the genes repressed by Pi starvation are more highly expressed in the *phr1 phl1* double mutant than in wild type after Pi starvation (Table 1). This indicates that a large proportion of the transcriptional repression response is also an integral part of the adaptive response, since it is evident that the *phr1 phl1* double mutant is more sensitive to Pi starvation, as it cannot mount a correct response (Figure 2B).

### Direct and indirect control of the molecular response to Pi starvation by PHR1(-like) TF

Here we show that PHR1(-like) regulation of Pi starvation-responsive genes involves both direct and indirect control. Direct control is essentially exerted on induced genes containing the P1BS (GNATATNC) sequence [12], whereas transcriptional repression is essentially indirect. Indeed, it can be noted that genes identified as direct targets (in which activation is independent of protein translation) are highly enriched in Pi starvation-induced genes containing P1BS sequences in different parts of the gene, particularly in the promoter proximal region and even to a higher extent in the 5′UTR. This indicates that PHR1 acts most prominently as a transcriptional activator, and that control of transcriptional repression is mostly, if not completely indirect (e.g., via activation of a transcriptional repressor). A large proportion of the Pi starvation-induced genes (more than 70%) are also probably controlled indirectly by PHR1, since only about 30% of Pi starvation-induced genes have a P1BS motif in their promoter proximal region, 5′UTR or 3′UTR, where P1BS content is significantly higher than in an average Arabidopsis gene.

Another finding is the strong association between P1BS content in the promoter and the degree of Pi starvation inducibility (Figure 4). It is interesting that although other regions are also P1BS-enriched, particularly the 5′UTR, but also the 3′UTR, introns and 1 kb proximal downstream region of Pi starvation-responsive genes, P1BS content in these regions does not correlate with inducibility. This suggests that the role of P1BS differs qualitatively in these regions compared to its role in the promoter.

The correlation between P1BS content in the promoter and gene inducibility is not strict, however; for *IPS1*, we show that one of the P1BS motifs in its promoter is in fact dispensable for Pi starvation responsiveness. In any case, the higher P1BS content of highly upregulated genes suggests that bioinformatic searches for stimulus-specific *cis*-regulatory motifs will be more efficient if performed in highly responsive genes.

Taken together, these observations suggest a simple evolutionary path to construct a complex adaptive response to a specific stress type, under the control of a central regulatory system. Our data are in agreement with a central regulator that controls pre-existing, shared genetic networks by acting on the regulators of those networks, as it is probably the case of *GSR* genes, rather than on each individual gene. In line with this idea, we found that in most cases, TF genes and non-TF genes are equally over-represented in the sets of genes responsive to Pi starvation and to any other type of stress (Table S3); we would predict under-representation of TF genes if shared genes were exclusively controlled by independent stress type-specific regulators. Genes for which the transcription rate obtained via this indirect route was insufficient, as could be the case of *RNS1* (see below), might have been recruited under the direct control of the central regulator, similar to the situation in Pi starvation-specific networks.

### Importance of P1BS *cis*-regulatory element as an integrator in the response to Pi starvation: concerted action *in vivo* with other *cis*-regulatory motifs

Here we demonstrate the key importance of P1BS in Pi starvation gene inducibility, reinforcing the importance of *PHR1*. In addition to the fact that P1BS is overrepresented in phosphate starvation-induced genes, as shown here and elsewhere [9], [11], P1BS is highly conserved in a Pi starvation-responsive gene (*IPS1*). Mutation of critical P1BS motifs in promoters of Pi-responsive genes abolishes Pi starvation responsiveness in dicots and monocots (Figure 5) [53], and a minimal promoter containing four tandem copies of P1BS is specifically responsive to Pi starvation (Figure 6).

The fact that a minimal promoter containing P1BS specifically responds to Pi starvation allowed us to examine the effect of several modulators of the Pi starvation response, and to show that this element can recapitulate Pi starvation-specific responsiveness (Figure 6); this includes the effect of all the best known modulators of this response, such as sugars, cytokinins, arsenate and long distance systemic repression [11], [46]–[50]. These data qualifys P1BS and, consequently, PHR1(-like) TF as central integrators of the Pi starvation response (Figure 6D).

By analysing the function of a promoter responsive to Pi starvation and wounding (*RNS1*) [43], we show that the P1BS motif is necessary only for Pi starvation responsiveness and not for responsiveness to other types of stress. Conversely, mutation of *PHR1* and *PHL1* affect only *RNS1* responsiveness to Pi starvation and not to other stress types (Figure 5). Independent multisignal responsiveness can thus also be attained through independent *cis* motifs in the promoter.

Although our data indicate the importance of P1BS as a Pi starvation response *cis* motif, we also show that P1BS function is dependent on sequence context, and that P1BS alone is insufficient to drive Pi starvation responsiveness in the context of a natural promoter such as that of *IPS1* (Figure 5 and Figure 7). Indeed, our phylogenetic and mutational analysis of *IPS1* identified a second motif, motif B (GAWGATNC), necessary for correct Pi starvation responsiveness of *IPS1*. The conditional overrepresentation of motif B, dependent on the presence of P1BS (Table S6), and the finding that motif B is unable to drive Pi starvation responsiveness strengthens the idea that PHR1 and P1BS represent a central integrator module in Pi starvation responsiveness.

### Conclusions

The results of this study show that *PHR1* and a functionally related member of its family comprise a central integrator system for the Pi starvation response. Pi limitation is a common condition in many natural soils, which implies that selective pressure against this stress has been very strong throughout evolution, underlining the adaptive value of this simple regulatory system of such a complex response. A consequence of our finding that a single TF family largely controls a stress response is that transcriptionally overlapping programs in response to different stress types can ultimately be controlled by independent regulatory systems. Such systems act indirectly, using (pre-existing) shared regulatory components in many targets, and directly on the remaining small proportion of target genes on average highly enriched in P1BS. The finding that the 5′UTR of PHR1 primary targets and of Pi starvation induced genes shows the highest overrepresentation in P1BS sequences, raises the possibility of an important role of this region in transcriptional control, in addition to its most commonly associated role in translational control. The fact that a large proportion of the transcriptionally repressed genes are controlled by PHR1(-like) TF indicates that transcriptional repression is an integral part of the Pi starvation response, and not merely a consequence of plant malfunction under stress.

## Materials and Methods

### Plant material and growth conditions

All *Arabidopsis thaliana* plants used in this study, including mutants and transgenic plants, were on the Columbia (Col-0) background. *phl1* was obtained from the SAIL collection (SAIL_731_B09) [54]. Growth conditions and the complete Johnson medium containing 2 mM Pi (KH_2_PO_4_) and 2% sucrose were as described [22], [55]. For specific experiments, the concentration of Pi, sucrose, kinetine or arsenate (NaH_2_AsO_4_·7H_2_O) is indicated.

### Physiological measurements

Anthocyanin was extracted from rosettes of plants grown on Pi-lacking medium supplemented with 5 µM DEX for 12 days. Anthocyanin content was measured as described [56]. The method of Ames [57] was used to determine the cellular phosphate content of seedlings grown on complete medium for 12 days (supplemented with 5 µM DEX when specified). Mean values were compared using Student's *t*-test.

### Constructs for expression in plants and plant transformation

Plants were transformed by the vacuum infiltration method [58]. Routine molecular work was performed as described [12], [59], except where indicated. Sequences of primers used for PCR amplification and construction of genomic DNA/cDNA fragments are given in Table S7.

A *Nco*I-*SpeI* fragment containing the ORF of *PHR1* was amplified by PCR from the *PHR1* cDNA [12] purified and digested with *NcoI* and *SpeI*. This fragment was introduced into the binary vector pBHAGR, which contains the CaMV 35S promoter, the 3xHA epitope and a fragment of the rat glucocorticoid receptor (GR) cDNA encoding the receptor-binding domain, generating the recombinant expression cassette *35S:HA:GR:PHR1* (pBHAGRPHR1). The pBHAGR vector was generated introducing a *Bam*HI*-Nco*I cDNA fragment codifying for the 277 carboxy-terminal amino acids of the rat glucocorticoid receptor [60] into a binary vector pBHA kindly supplied by Dr. F. Parcy (Institut National de la Recherche Agronomique, Grenoble, France).

The *Hin*dIII-*Bam*HI 1kb fragment containing the *IPS1* promoter and the *Xba*I*-blunt* 1kb fragment containing the *RNS1* promoter were amplified by PCR. The mutated promoter sequences were generated as overlapping PCR products using semi-complementary primers with the mutated sequences. The PCR products were purified, digested with *Hin*dIII-*Bam*HI (*IPS1*) or *Xba*I (*RNS1*) and inserted between *Hin*dIII-*Bam*HI or *Xba*I-*Sma*I sites into the pBI101 vector [44].

The four tandem copies of P1BS (*4xP1BS:GUS*), the B motif (*4xB:GUS*) constructs, and the 42bp *IPS1* promoter fragment (*A-P1BS-B:GUS*) were generated by annealing semi-complementary primers, resulting in DNA fragments with *Hin*dIII and *Xba*I overhangs. The over-hanged DNA fragments were inserted between *Hin*dIII and *Xba*I sites at the 5′ end of a minimal 35S promoter in the pTi0046 plasmid. The pTi0046 plasmid contains a −46bp truncated version of the CaMV 35S promoter [45] into the *Bam*HI site of the pBI101 vector.

### Real-Time PCR

Quantitative PCR (Q-RT-PCR) was performed on three independent biological samples as described [61]. The pairs of primers used are described in Table S7.

### Protein synthesis, DNA binding reactions and EMSA analysis

PHR1 and PHL1 deletion derivatives were generated by *in vitro* translation (or cotranslation in the dimerization experiments) using the TnT T7 Quick System for PCR DNA (Promega), as described [62]. PCR and labeling of promoter fragments and oligonucleotides, DNA binding reactions and EMSA were performed as described [63].

### Yeast two-hybrid assays

A cDNA fragment corresponding to a deletion derivative of PHR1, Δ-PHR1, encompassing amino acid residues 208–362, that lacks transactivation domain was cloned into the pGBKT7 (Gal4 DNA binding domain, BD; Clontech). We used this to screen a whole seedling cDNA library prepared in the pGADT7 vector (Gal4 activation domain, AD, Clontech) to detect PHR1-interacting proteins. One of these was Δ-PHL1 (lacking amino acids 1–60, details to be described elsewhere). To confirm protein interactions, the plasmids were cotransformed in *Saccharomyces cerevisiae* AH 109 cells following standard heat-shock protocols [64]. Successfully transformed colonies were identified on yeast synthetic drop-out lacking Leu and Trp; these colonies were resuspended in 30 mM NaCl and transferred to the same media plus β-gal or to selective media lacking Ade, His, Leu and Trp. Plates were incubated (30°C, 2–4 days). The empty vector pGADT7 was also cotransformed with the pGBKT7-DPHR1 construct as a negative control.

### Transcriptome analyses of Pi starvation response and of PHR1 direct targets

Transcriptomic analyses were performed using the Affymetrix ATH1 platform. For the phosphate starvation response analysis, wt, *phr1* and *phr1 phl1* plants were grown for 7 days in complete (+Pi) or phosphate-lacking (−Pi) solid media, and roots and shoots were processed separately. For PHR1 direct target analysis, complete *OxGR:PHR1 phr1* and *phr1* plants were grown for 7 days in +Pi liquid media, then for 2 days in −Pi liquid media and harvested after 6 h treatment with 5 µM DEX and 10 µM CHX. In each experiment, RNA was isolated from three independent biological samples using the RNeasy plant mini kit (Quiagen). Biotin-labeled cRNA was synthesized using One-Cycle target labelling and control reagents (Affymetrix, Santa Clara, CA) and fragmented into 35–200 bases in length. Three replicates for each condition were hybridized independently to the Arabidopsis ATH1 Genome array following manufacturer's recommendations (Affymetrix). Each microarray was washed and stained with streptavidin-phycoerythrin and scanned at 2.5 µm resolution in a GeneChip Scanner 3000 7G System (Affymetrix). Data analyses were performed using GeneChip Operating Software (GCOS) and analyzed using the affylmGUIR package [65]. Robust Muti-array Analysis (RMA) algorithm was used for background correction, normalization and expression levels summarization [66]. Differential expression analysis was performed with the Bayes t-statistics from the linear models for Microarray data (limma). P-values were corrected for multiple-testing using the Benjamini-Hochberg method (False Discovery Rate) [67]. Except where indicated, genes were considered to be differentially expressed if corrected P values were <0.05, and only genes with a signal log ratio more than one or less than minus one were considered for further analysis.

### Transcriptome comparisons

For transcriptome comparisons we used microarray data for different treatments/stresses available in the GENEVESTIGATOR database (https://www.genevestigator.com) [30]. The two-fold up- and down-regulated genes were identified by the Meta-Analyser tool included in this platform. Transcription factor genes for transcriptomic analysis were identified in the RARTF Database (http://rarge.psc.riken.jp/rartf/) [68].

### Mapman ontology display

Average expression signals for the Pi starvation treatment were expressed relative to those in complete media, converted to a log_2_ scale and imported into the MapMan software, which showed values in colour scale diagrams (http://mapman.gabipd.org/web/guest/home) [35].

### Isolation of promoter regions

The promoters regions of gene orthologs were obtained using commercially available GenomeWalker technology (Clontech), following manufacturer's recommendations. Sequences of interest were obtained by two rounds of PCR amplification using as template the adapter-ligated genomic DNA from different *Brassicaceae* species, an *IPS1*-specific primer and the adaptor primer. Primary PCR was performed with seven cycles of 25 sec at 94°C and 4 min at 72°C, followed by 32 cycles of 25 sec at 94°C and 4 min at 67°C, with a final extension of 4 min at 67°C. Secondary PCR was performed using a 1∶50 dilution of the primary reaction product as a template and similar PCR cycling parameters, with 5 and 22 cycles of the first and second steps, respectively. PCR products were cloned into the pCRII-TOPO TA system (Invitrogen). Sequences were aligned using DiAlign (http://www.genomatix.de/cgi-bin/dialign/dialign.pl) [69].

### Phylogenetic analysis of the MYB-CC family

The analysis was performed on the Phylogeny.fr platform (www.phylogeny.fr) [70]. Sequences were aligned with MUSCLE (v3.7) configured for highest accuracy. After alignment, ambiguous regions were removed with Gblocks (v0.91b). The phylogenetic tree was reconstructed using PhyML program (v3.0 aLRT). The default substitution model was selected assuming an estimated proportion of invariant sites (of 0.021) and 4 gamma-distributed rate categories to account for rate heterogeneity across sites. The gamma shape parameter was estimated directly from the data (gamma = 1.044). Reliability for internal branch was assessed using the bootstrapping method (100 bootstrap replicates). The tree was represented withTreeDyn (v198.3).

### Accession Numbers

Arabidopsis Genome Initiative locus identifiers for the genes mentioned in this article are At5g29000 (*PHL1*), At4g28610 (*PHR1*), At5g43350 (*PHT1*;1), At2g02990 (*RNS1*), At3g09922 (*IPS1*), At5g20150 (*SPX1*), At5g03545 (*At4*), At4g33030 (*SQD1*) and At3g17790 (*ACP5*). The GenBank accession numbers for the sequences of the proximal promoter region of *IPS1* orthologs are GQ184774 (*Descurainia sophia*, *DsIPS1*), GQ184775 (*Arabis auriculata*, *AaIPS1*), GQ184776 (*Brassica intermedia*, *BiIPS1*) and GQ184777 (*Lepidium campestre*, *LcIPS1*). The GEO accession number for the array experiments are GSE16722 and GSE20955.

## Supporting Information

Figure S1 Sequence comparison of MYB-CC family TF members from Arabidopsis. Sequence alignment was done with MUSCLE (v3.7) configured for highest accuracy using the Phylogeny.fr platform (www.phylogeny.fr) [69]. In addition to the AGI number, names are given for the functionally characterized members: PHOSPHATE STARVATION RESPONSE REGULATOR 1 (PHR1) [12]; PHR1-LIKE1 (PHL1; this study) ALTERED PHLOEM DEVELOPMENT (APL) [70]. Only sequences of the conserved MYB (top) and coiled-coil (CC; lower part) domains were considered. Amino acids conserved among family members are shown (black boxes); amino acids identical to PHR1 (grey). For each protein, the percentage of amino acid identity to PHR1 in the MYB and CC domains is shown (right; % aa identity).
(3.70 MB TIF)

Figure S2 Characterisation of *phl1* insertional mutant. (A) Scheme shows *PHL1* and the site of T-DNA insertion in *phl1* (top) and semiquantitative RT-PCR expression analysis of *PHR1* (R1) and *PHL1* (L1) in wild type (wt), and *phr1* and *phl1* mutants (bottom). Plants were grown for 7 days in −Pi media before harvest. Oligonucleotides for *PHL1* expression analyses flank the T-DNA insertion site. (B) Northern analysis of *PHR1* and *PHL1* expression. Plants were grown for 7 days in +Pi or −Pi media, and northern blots were sequentially hybridised to the *PHL1* and *PHR1* probes. (C) *PHR1* and *PHL1* expression at different developmental stages according to GENEVESTIGATOR database (https://www.genevestigator.com) [30].
(1.05 MB TIF)

Figure S3 Expression of Pi starvation induced genes on wt, *phr1*, *phl1* and *phr1phl1* plants grown in a Pi-rich regimen. Quantitative RT-PCR was performed on cDNA prepared from RNA corresponding to three independent biological samples of wild type (wt), *phr1*, *phr1phl1* and *phl1* plants grown for 7 days in Pi-rich medium.
(0.44 MB TIF)

Figure S4 Northern analysis of expression of Pi starvation-responsive genes in GR:PHR1 overexpressing lines. Wild type (wt), *phr1* and PHR1 overexpressing (OxPHR1) plants were grown for 7 days in +Pi or −Pi media alone or supplemented with 5 µM DEX (+DEX). RNA from roots and shoots was isolated separately and northern blots were sequentially hybridised to the probes *PHT1;1*, *IPS1*, *SPX1* and *PHR1*. Ethidium bromide-stained rRNA was used as loading control.
(0.45 MB TIF)

Figure S5 Interaction of PHR1 and PHL1 proteins in the yeast two-hybrid assay. Yeast cells co-transformed with pGBKT7-ΔPHR1 (BD-PHR1), expressing a PHR1 deletion derivative encompassing amino acid residues 208–362 fused to the GAL4 DNA-binding domain and pGADT7-ΔPHL1 (AD-PHL1), expressing a PHL1 deletion lacking amino acid residues 1–60, were selected on yeast synthetic drop-out medium lacking Trp and Leu (−WL), then transferred to the same media plus β-gal or to selective media lacking Trp, Leu, His and Ade (−WLHA) to test protein interactions. pGBKT7-ΔPHR1 cotransformation with empty pGADT7 vector (AD∅) was included as a control.
(0.40 MB TIF)

Figure S6 Dendogram showing the distribution of genes whose expression is altered in *phr1* and *phr1 phl1 versus* wild type plants grown in −Pi medium, according to their Pi starvation responsiveness. Arabidopsis genes represented in the ATH1 affymetix microarray were classified according to their Pi starvation responsiveness in wild type plants. Pi starvation induced, expression ratio in plants grown in −Pi *versus* +Pi conditions>1.1×; High (>4×, H), Medium (2–4×, M), Low (2–1.5×, L), Very Low (1.5–1.1×, VL); Non Pi starvation responsive (NR), expression ratio in plants grown in −Pi *versus* +Pi conditions between 1.1–0.9×; Pi starvation repressed, expression ratio in plants grown in −Pi *versus* +Pi conditions <0.9×; High (<0.25×, H), Medium (0.25–0.5×, M), Low (0.5–0.66×, L), Very Low (0.66–0.9×, VL). The dendogram shows the percentage of the genes in each class whose expression is altered by the *phr1* and *phr1 phl1* mutations (2×, FDR<0.05).
(0.48 MB TIF)

Figure S7 Differential expression of genes involved in metabolism (A) and regulation (B) in the Pi starvation response. Transcript levels in shoot (top) and root (bottom) from plants grown in −Pi conditions relative to those of plants grown in +Pi medium. Results are the mean of three replicates, displayed on a log_2_ scale using MapMan software [35]. Transcripts that increase and decrease are shown by an increasingly intense blue and red colours, respectively. A scale was selected in which values of 0.2 and 1 on a log_2_ scale gave faint and full saturation, respectively. The data can be explored interactively by downloading the experimental data files and MapMan software from http://mapman.gabipd.org/web/guest/home [35].
(3.63 MB TIF)

Figure S8 Relationship between P1BS content in different parts of Pi starvation-induced genes, and inducibility and specificity. (A) Average number of other stresses in which Pi starvation-induced genes are also induced, relative to the number of P1BS motifs present in different parts of the gene. Data for induction by other stress types were obtained from 28 stress conditions for which transcriptomic data were available in the GENEVESTIGATOR database (https://www.genevestigator.com) [30]. Asterisks represent significant differences (p<0.01 using the Χ^2^ test). (B) Relation between the number of P1BS motifs in different parts of the gene and log_2_ x-fold induction. The number of P1BS/gene (No P1BS/gene) was calculated as the average content of P1BS motifs over successive sets of 30 genes, measured at a one-gene interval, ordered according to inducibility by Pi starvation.
(0.54 MB TIF)

Figure S9 Sequence comparison of the proximal promoter regions of IPS1 orthologous genes from different Brasicaceae species. Sequences of the proximal promoter regions of *IPS1* orthologous genes from five *Brassicaceae* species (*Arabidopsis thaliana*, *AtIPS1*; *Descurainia sophia*, *DsIPS1*; *Arabis auriculata*, *AaIPS1*; *Brassica intermedia*, *BiIPS1*; *Lepidium campestre*, *LcIPS1*) were aligned using the DiAlign software (http://www.genomatix.de/cgi-bin/dialign/dialign.pl) [68]. Large-size conserved regions among IPS1 orthologues are shadowed in grey. Sequences shared with *At4* are highlighted in yellow, and relevant motifs are indicated.
(3.10 MB TIF)

Table S1 Transcriptomic data of the Pi starvation response in wild type, *phr1* and *phr1 phl1* mutants.
(10.34 MB XLS)

Table S2 Genes showing altered expression in Pi starved *phr1 phl1 versus phr1*.
(0.39 MB TIF)

Table S3 Overlaps among transcriptional responses to Pi starvation and to other stimuli/conditions.
(2.36 MB TIF)

Table S4 Transcriptomic data of *OxGR:PHR1 phr1 vs. phr1* in Pi starvation conditions.
(3.23 MB XLS)

Table S5 Distribution of P1BS sequences in different parts of PHR1 direct target genes and of Pi starvation-responsive genes.
(1.48 MB TIF)

Table S6 Overrepresentation of closely linked P1BS and B motifs in Pi starvation-induced genes.
(1.47 MB TIF)

Table S7 Primers used in this study.
(0.81 MB TIF)
