# Supplementary figures and images for: A Central Regulatory System Largely Controls Transcriptional Activation and Repression Responses to Phosphate Starvation in Arabidopsis
Source: PLoS Genet. 2010 Sep 9;6(9):e1001102. doi: 10.1371/journal.pgen.1001102 (PMC2936532; doi:10.1371/journal.pgen.1001102)

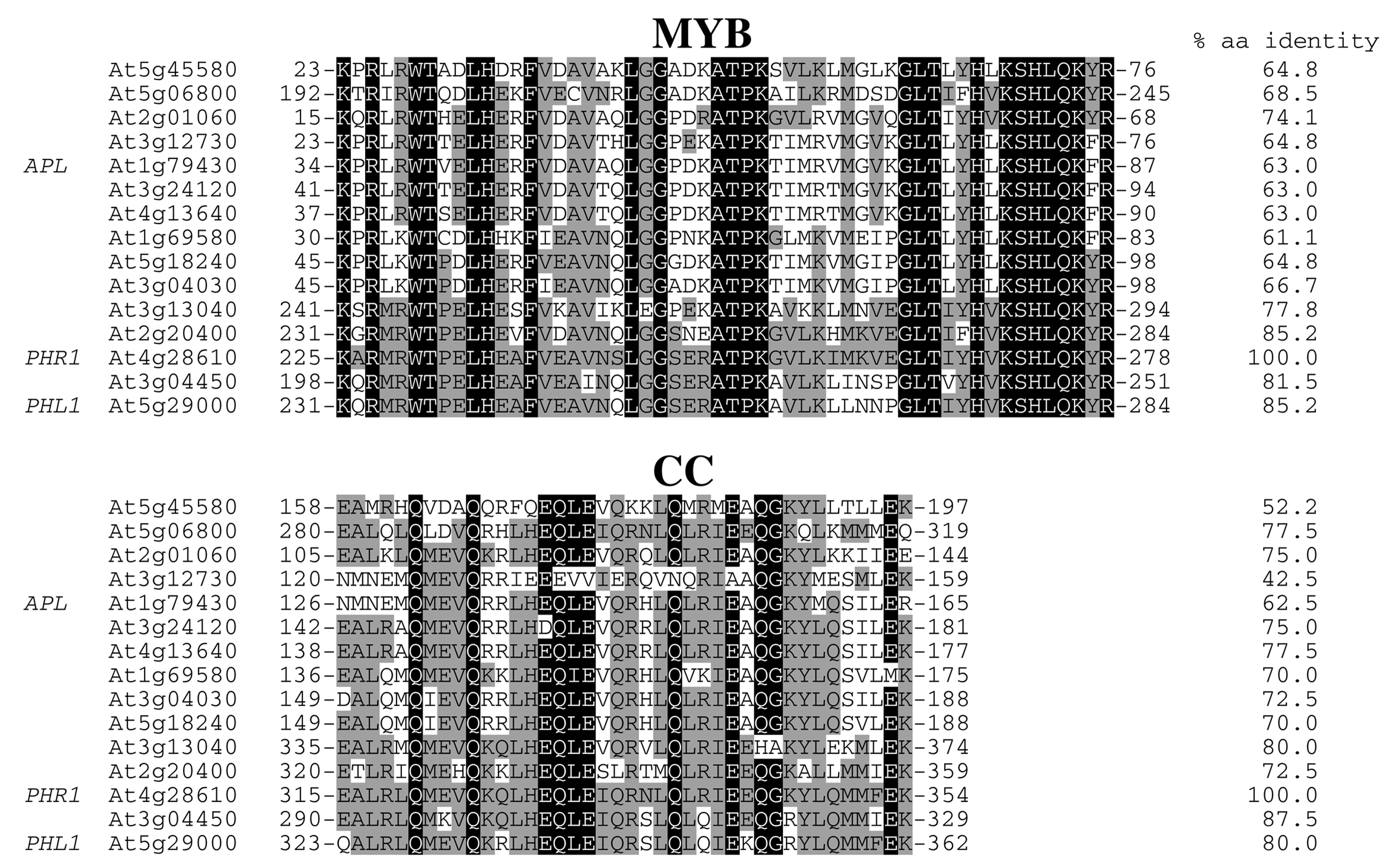

Supplement: Figure S1 — Sequence comparison of MYB-CC family TF members from Arabidopsis. Sequence alignment was done with MUSCLE (v3.7) configured for highest accuracy using the Phylogeny.fr platform (www.phylogeny.fr) [69]. In addition to the AGI number, names are given for the functionally characterized members: PHOSPHATE STARVATION RESPONSE REGULATOR 1 (PHR1) [12]; PHR1-LIKE1 (PHL1; this study) ALTERED PHLOEM DEVELOPMENT (APL) [70]. Only sequences of the conserved MYB (top) and coiled-coil (CC; lower part) domains were considered. Amino acids conserved among family members are shown (black boxes); amino acids identical to PHR1 (grey). For each protein, the percentage of amino acid identity to PHR1 in the MYB and CC domains is shown (right; % aa identity). (3.70 MB TIF) [file pgen.1001102.s001.tif]

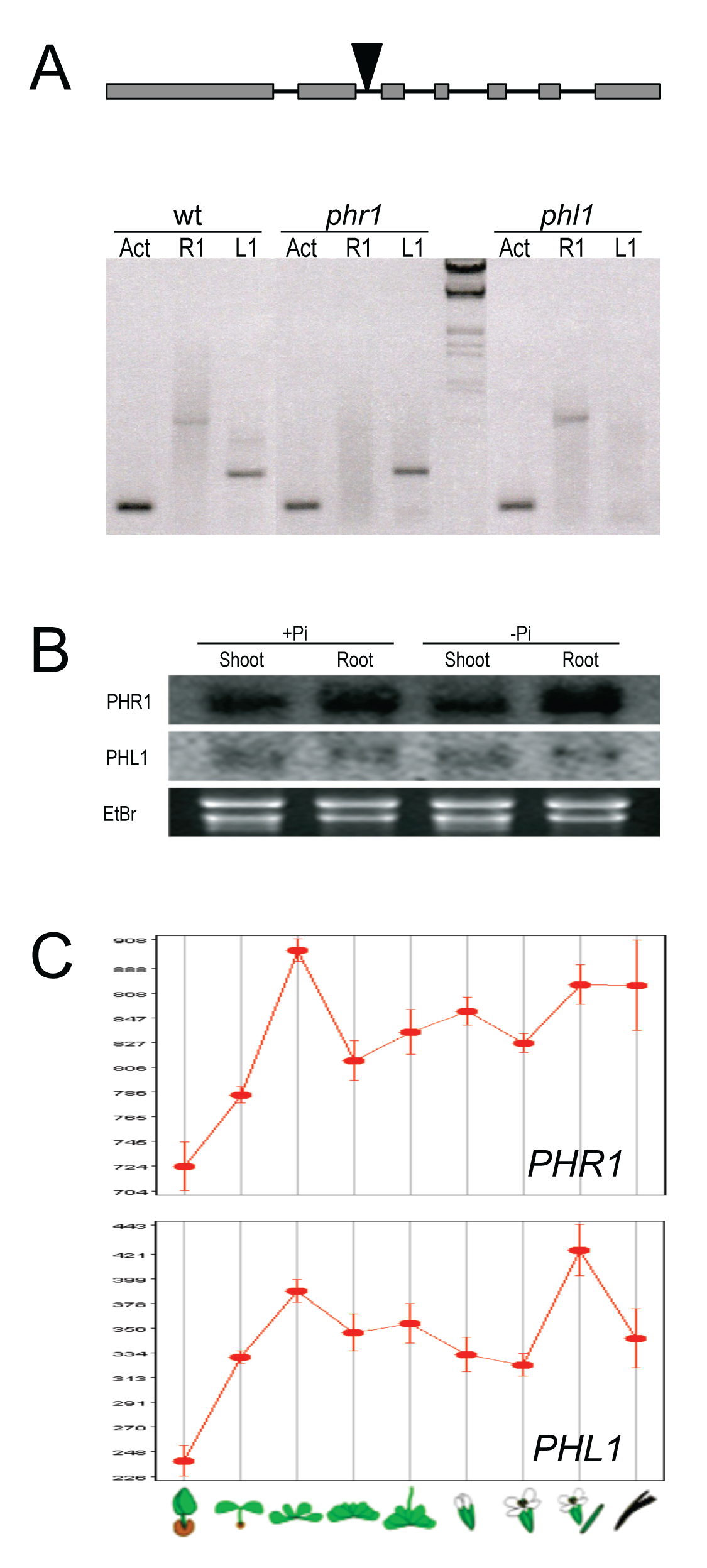

Supplement: Figure S2 — Characterisation of phl1 insertional mutant. (A) Scheme shows PHL1 and the site of T-DNA insertion in phl1 (top) and semiquantitative RT-PCR expression analysis of PHR1 (R1) and PHL1 (L1) in wild type (wt), and phr1 and phl1 mutants (bottom). Plants were grown for 7 days in −Pi media before harvest. Oligonucleotides for PHL1 expression analyses flank the T-DNA insertion site. (B) Northern analysis of PHR1 and PHL1 expression. Plants were grown for 7 days in +Pi or −Pi media, and northern blots were sequentially hybridised to the PHL1 and PHR1 probes. (C) PHR1 and PHL1 expression at different developmental stages according to GENEVESTIGATOR database (https://www.genevestigator.com) [30]. (1.05 MB TIF) [file pgen.1001102.s002.tif]

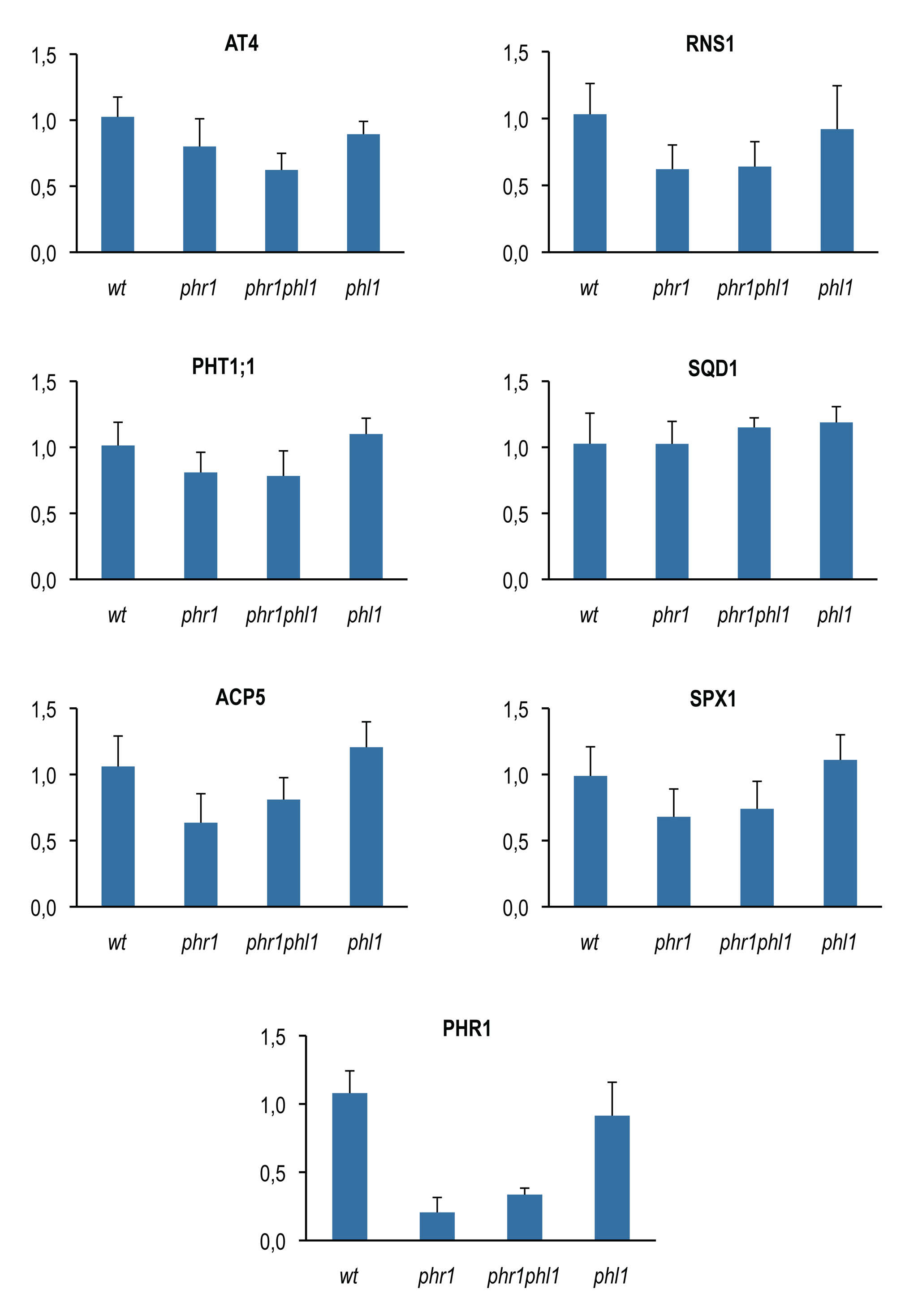

Supplement: Figure S3 — Expression of Pi starvation induced genes on wt, phr1, phl1 and phr1phl1 plants grown in a Pi-rich regimen. Quantitative RT-PCR was performed on cDNA prepared from RNA corresponding to three independent biological samples of wild type (wt), phr1, phr1phl1 and phl1 plants grown for 7 days in Pi-rich medium. (0.44 MB TIF) [file pgen.1001102.s003.tif]

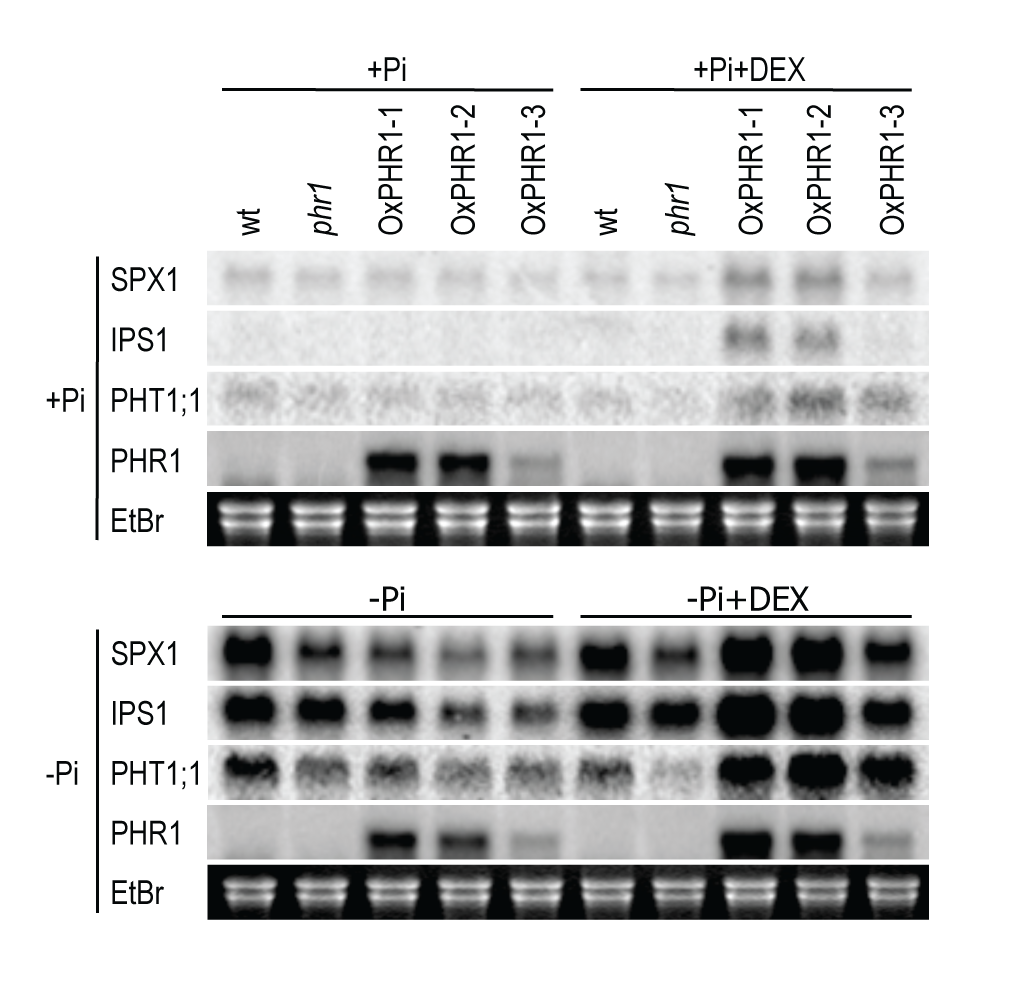

Supplement: Figure S4 — Northern analysis of expression of Pi starvation-responsive genes in GR:PHR1 overexpressing lines. Wild type (wt), phr1 and PHR1 overexpressing (OxPHR1) plants were grown for 7 days in +Pi or −Pi media alone or supplemented with 5 µM DEX (+DEX). RNA from roots and shoots was isolated separately and northern blots were sequentially hybridised to the probes PHT1;1, IPS1, SPX1 and PHR1. Ethidium bromide-stained rRNA was used as loading control. (0.45 MB TIF) [file pgen.1001102.s004.tif]

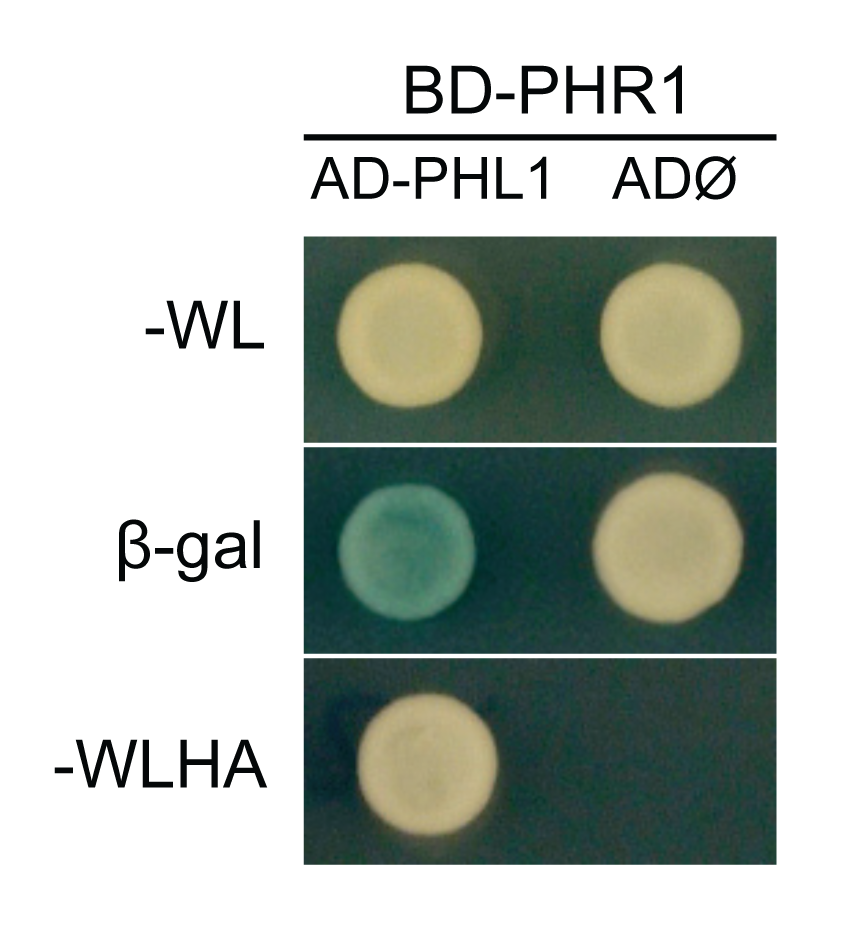

Supplement: Figure S5 — Interaction of PHR1 and PHL1 proteins in the yeast two-hybrid assay. Yeast cells co-transformed with pGBKT7-ΔPHR1 (BD-PHR1), expressing a PHR1 deletion derivative encompassing amino acid residues 208–362 fused to the GAL4 DNA-binding domain and pGADT7-ΔPHL1 (AD-PHL1), expressing a PHL1 deletion lacking amino acid residues 1–60, were selected on yeast synthetic drop-out medium lacking Trp and Leu (−WL), then transferred to the same media plus β-gal or to selective media lacking Trp, Leu, His and Ade (−WLHA) to test protein interactions. pGBKT7-ΔPHR1 cotransformation with empty pGADT7 vector (AD∅) was included as a control. (0.40 MB TIF) [file pgen.1001102.s005.tif]

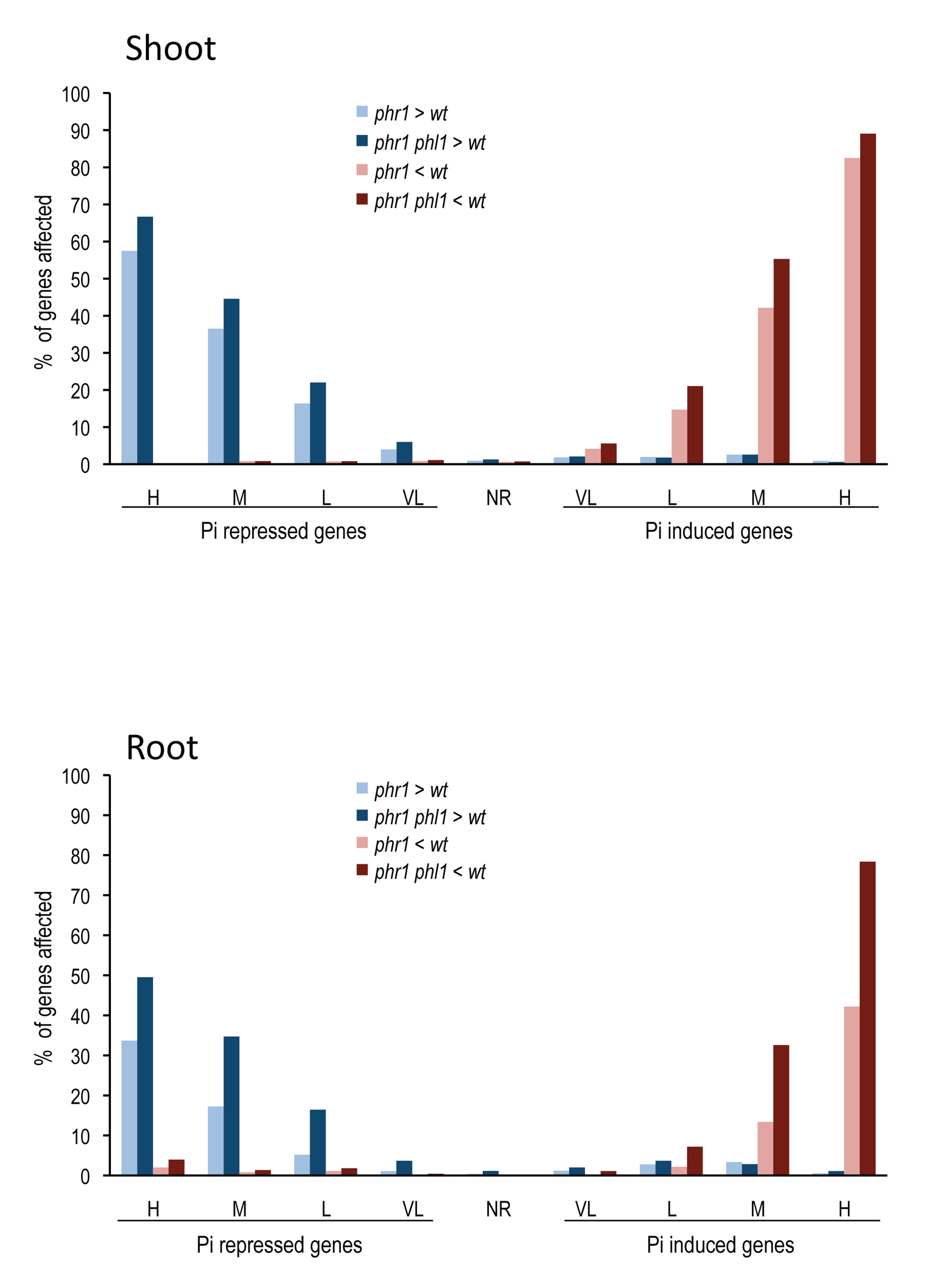

Supplement: Figure S6 — Dendogram showing the distribution of genes whose expression is altered in phr1 and phr1 phl1 versus wild type plants grown in −Pi medium, according to their Pi starvation responsiveness. Arabidopsis genes represented in the ATH1 affymetix microarray were classified according to their Pi starvation responsiveness in wild type plants. Pi starvation induced, expression ratio in plants grown in −Pi versus +Pi conditions>1.1×; High (>4×, H), Medium (2–4×, M), Low (2–1.5×, L), Very Low (1.5–1.1×, VL); Non Pi starvation responsive (NR), expression ratio in plants grown in −Pi versus +Pi conditions between 1.1–0.9×; Pi starvation repressed, expression ratio in plants grown in −Pi versus +Pi conditions <0.9×; High (<0.25×, H), Medium (0.25–0.5×, M), Low (0.5–0.66×, L), Very Low (0.66–0.9×, VL). The dendogram shows the percentage of the genes in each class whose expression is altered by the phr1 and phr1 phl1 mutations (2×, FDR<0.05). (0.48 MB TIF) [file pgen.1001102.s006.tif]

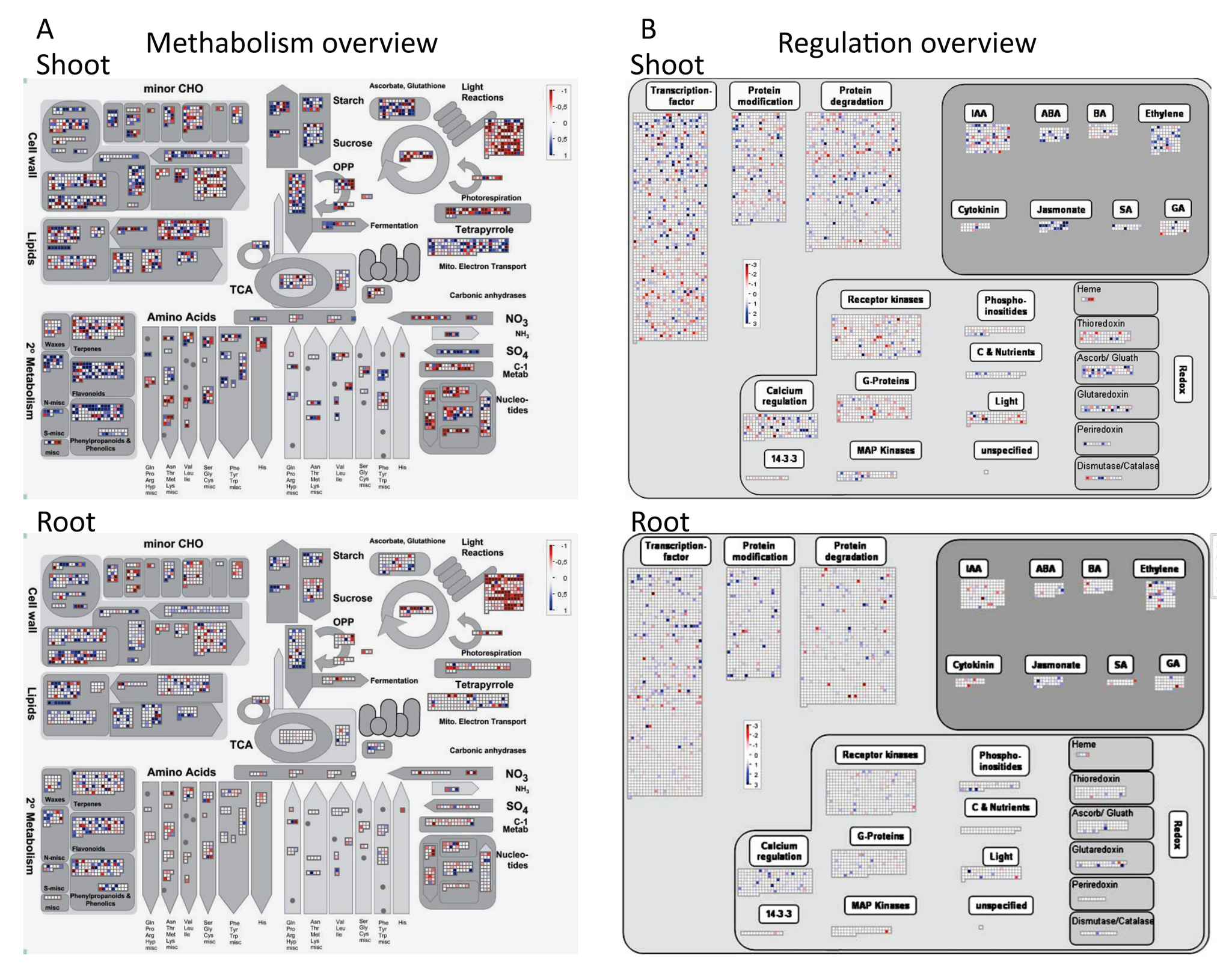

Supplement: Figure S7 — Differential expression of genes involved in metabolism (A) and regulation (B) in the Pi starvation response. Transcript levels in shoot (top) and root (bottom) from plants grown in −Pi conditions relative to those of plants grown in +Pi medium. Results are the mean of three replicates, displayed on a log2 scale using MapMan software [35]. Transcripts that increase and decrease are shown by an increasingly intense blue and red colours, respectively. A scale was selected in which values of 0.2 and 1 on a log2 scale gave faint and full saturation, respectively. The data can be explored interactively by downloading the experimental data files and MapMan software from http://mapman.gabipd.org/web/guest/home [35]. (3.63 MB TIF) [file pgen.1001102.s007.tif]

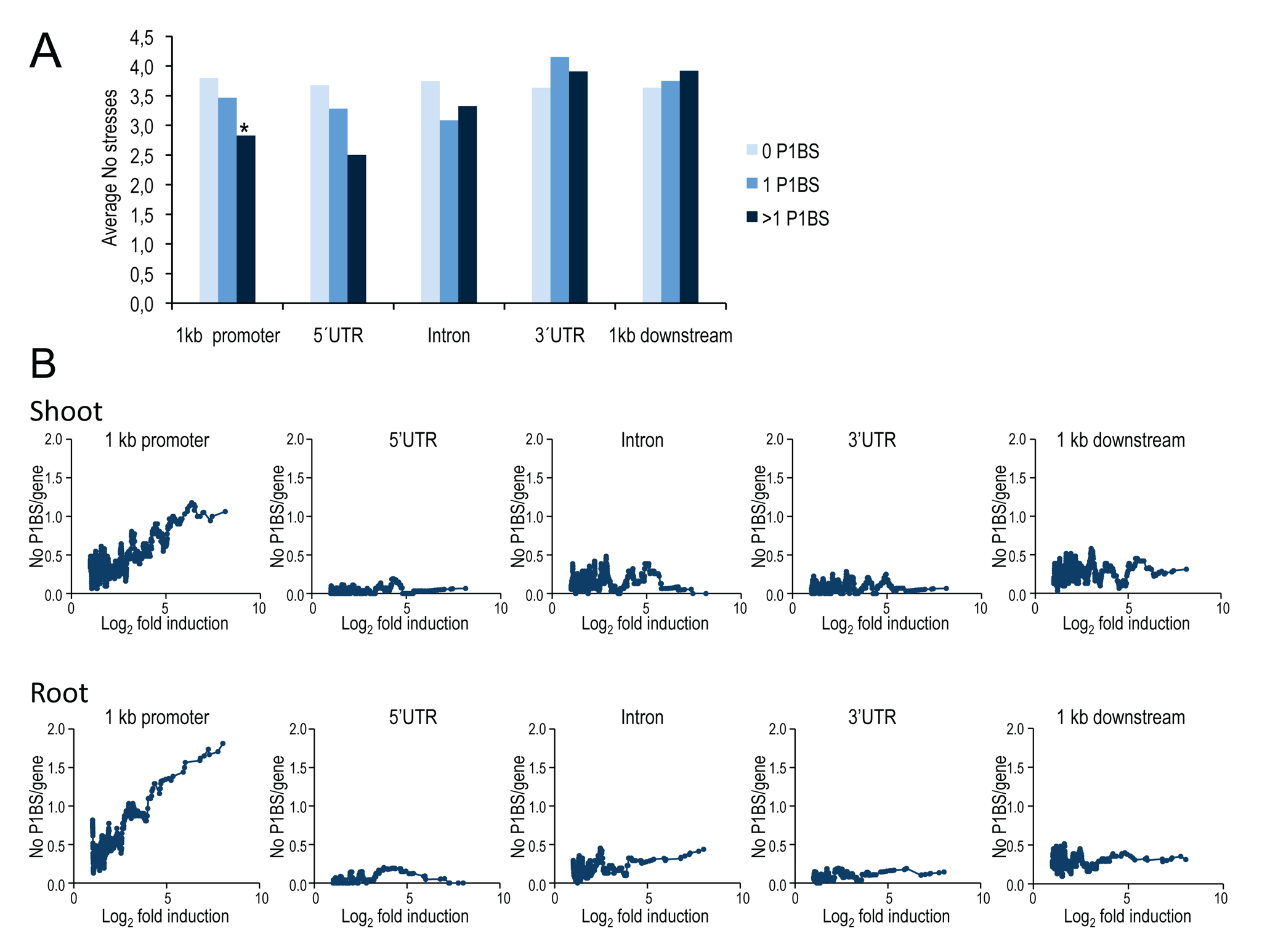

Supplement: Figure S8 — Relationship between P1BS content in different parts of Pi starvation-induced genes, and inducibility and specificity. (A) Average number of other stresses in which Pi starvation-induced genes are also induced, relative to the number of P1BS motifs present in different parts of the gene. Data for induction by other stress types were obtained from 28 stress conditions for which transcriptomic data were available in the GENEVESTIGATOR database (https://www.genevestigator.com) [30]. Asterisks represent significant differences (p<0.01 using the Χ2 test). (B) Relation between the number of P1BS motifs in different parts of the gene and log2 x-fold induction. The number of P1BS/gene (No P1BS/gene) was calculated as the average content of P1BS motifs over successive sets of 30 genes, measured at a one-gene interval, ordered according to inducibility by Pi starvation. (0.54 MB TIF) [file pgen.1001102.s008.tif]

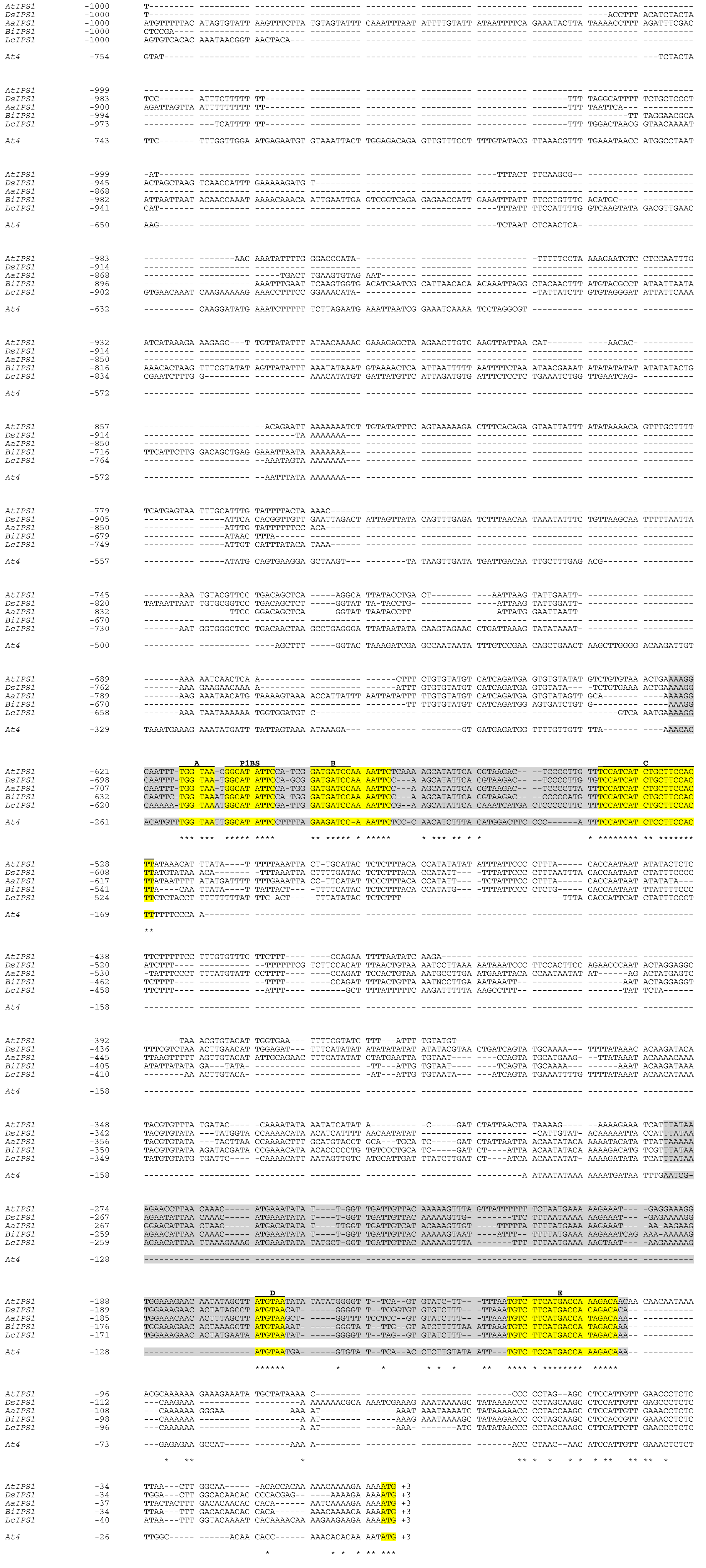

Supplement: Figure S9 — Sequence comparison of the proximal promoter regions of IPS1 orthologous genes from different Brasicaceae species. Sequences of the proximal promoter regions of IPS1 orthologous genes from five Brassicaceae species (Arabidopsis thaliana, AtIPS1; Descurainia sophia, DsIPS1; Arabis auriculata, AaIPS1; Brassica intermedia, BiIPS1; Lepidium campestre, LcIPS1) were aligned using the DiAlign software (http://www.genomatix.de/cgi-bin/dialign/dialign.pl) [68]. Large-size conserved regions among IPS1 orthologues are shadowed in grey. Sequences shared with At4 are highlighted in yellow, and relevant motifs are indicated. (3.10 MB TIF) [file pgen.1001102.s009.tif]

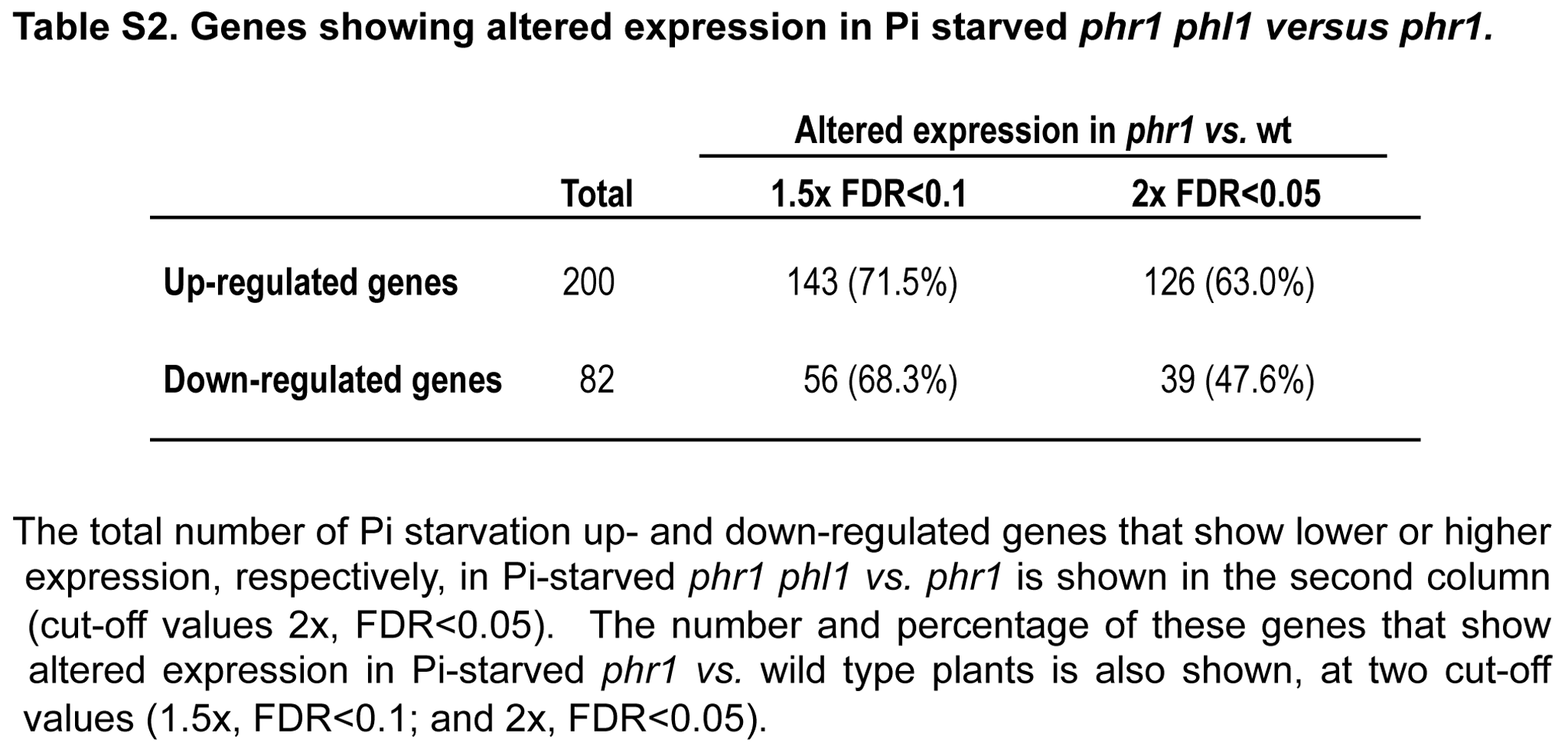

Supplement: Table S2 — Genes showing altered expression in Pi starved phr1 phl1 versus phr1. (0.39 MB TIF) [file pgen.1001102.s011.tif]

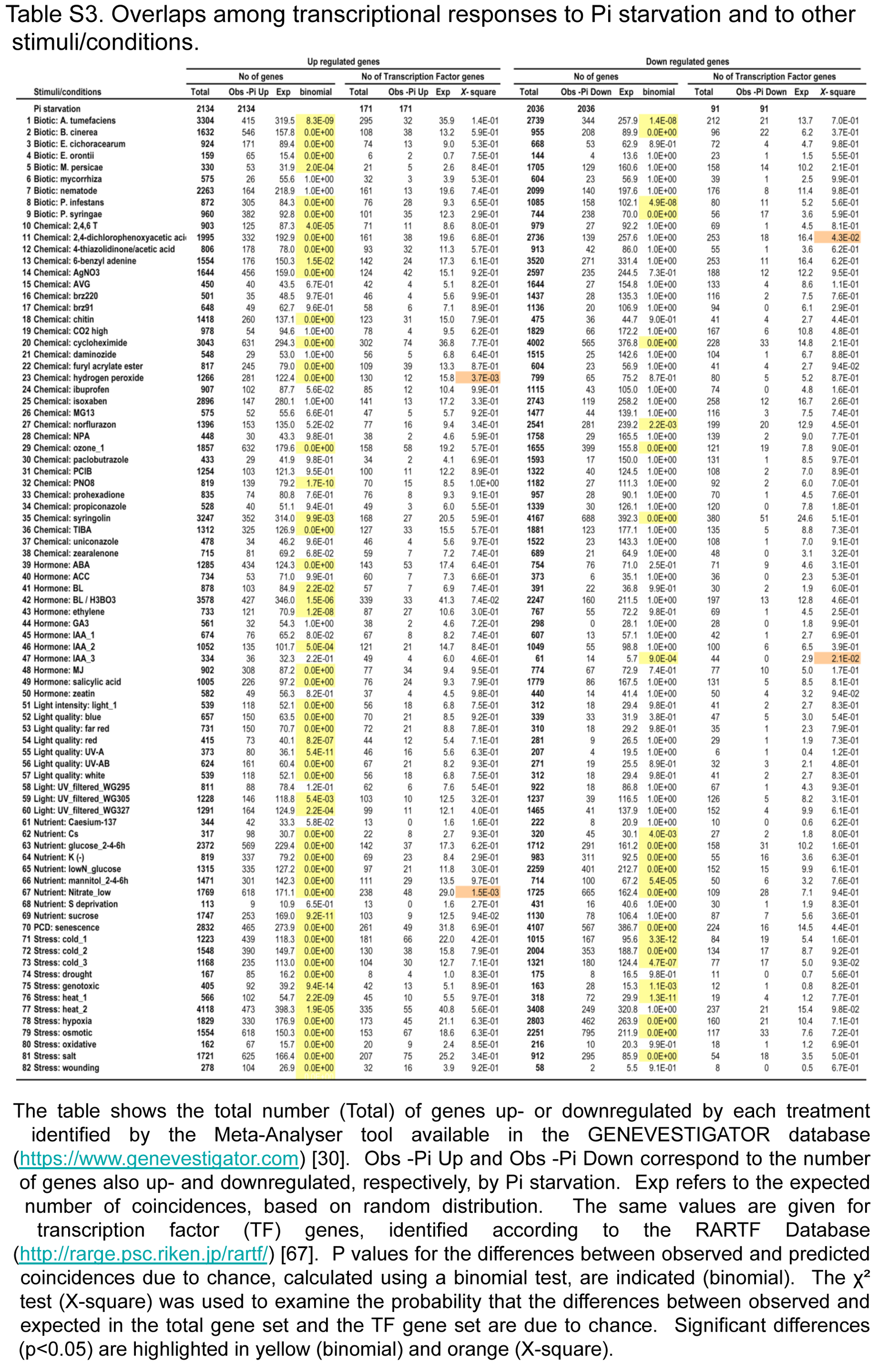

Supplement: Table S3 — Overlaps among transcriptional responses to Pi starvation and to other stimuli/conditions. (2.36 MB TIF) [file pgen.1001102.s012.tif]

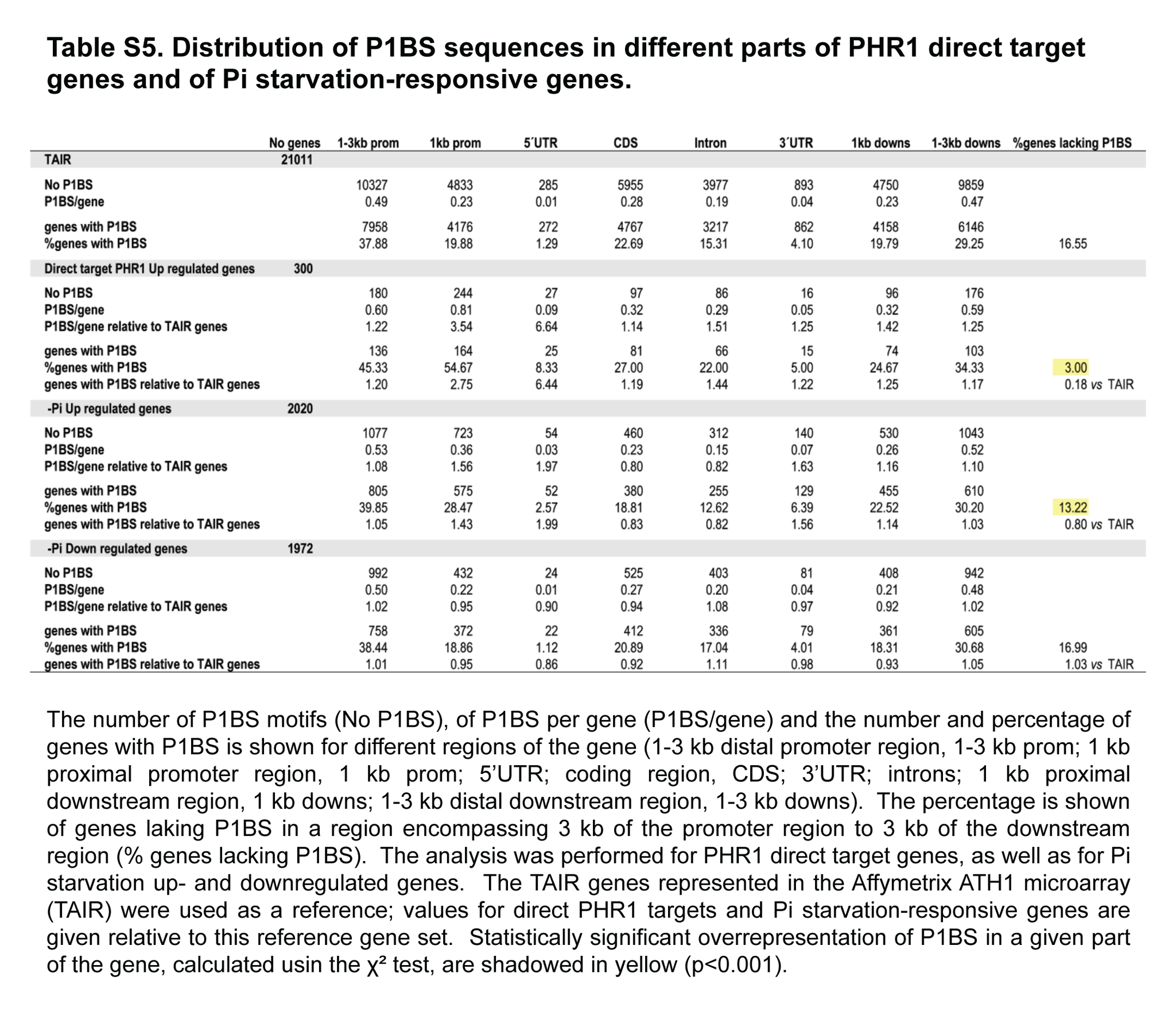

Supplement: Table S5 — Distribution of P1BS sequences in different parts of PHR1 direct target genes and of Pi starvation-responsive genes. (1.48 MB TIF) [file pgen.1001102.s014.tif]

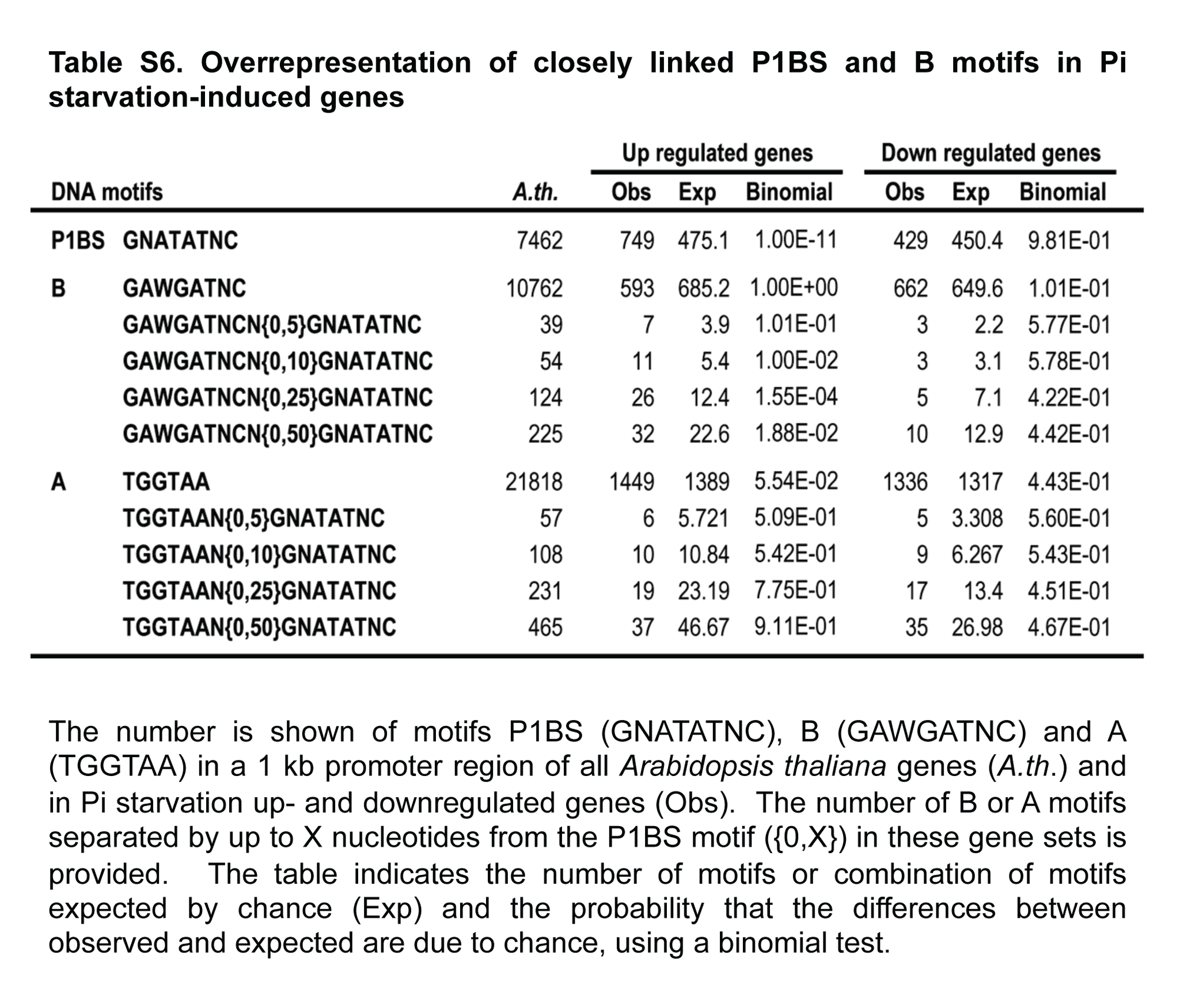

Supplement: Table S6 — Overrepresentation of closely linked P1BS and B motifs in Pi starvation-induced genes. (1.47 MB TIF) [file pgen.1001102.s015.tif]

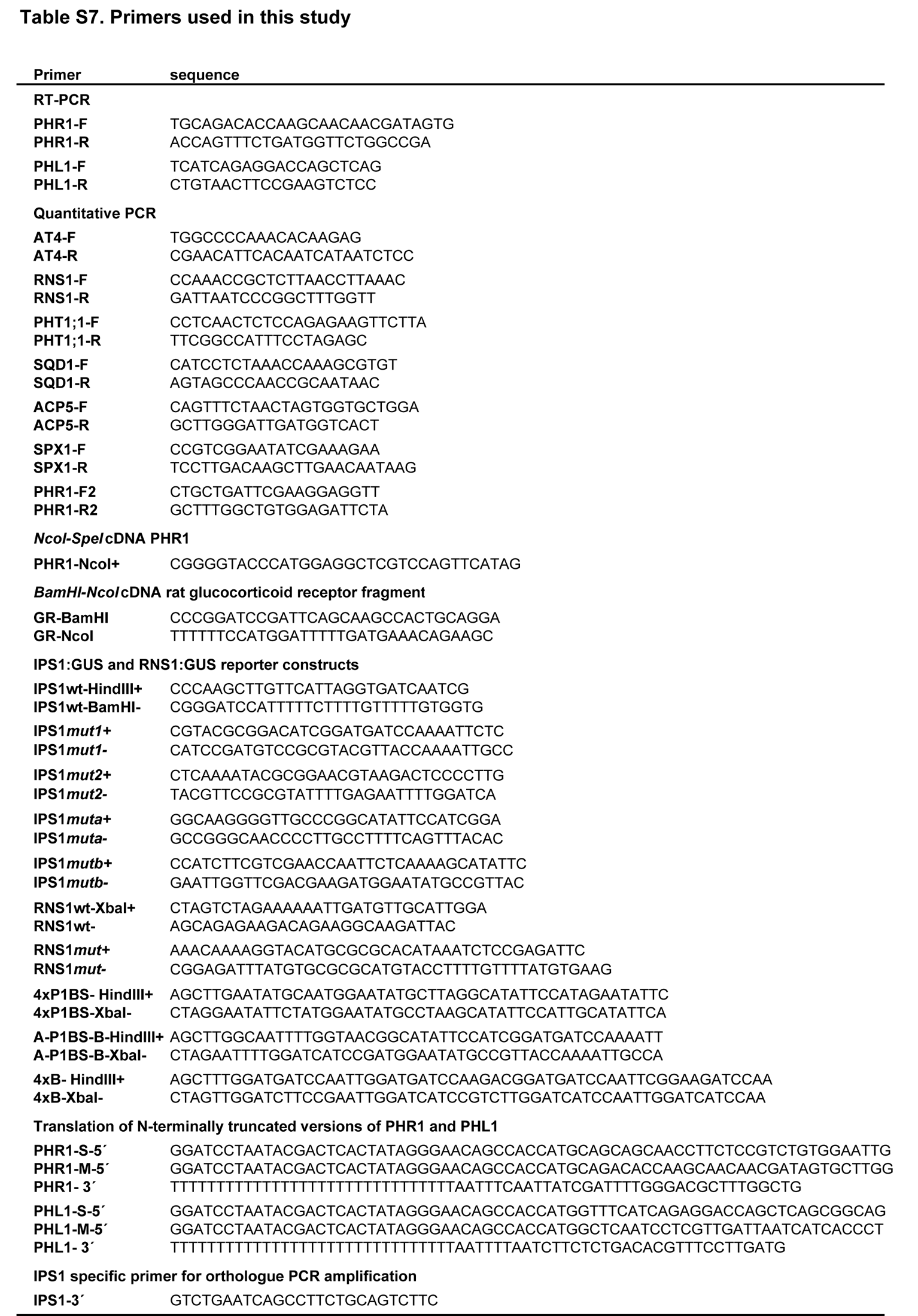

Supplement: Table S7 — Primers used in this study. (0.81 MB TIF) [file pgen.1001102.s016.tif]
